# A Comparative Review of the SWEET Simulator: Theoretical Verification Against Other Simulators

**DOI:** 10.3390/jimaging10120306

**Published:** 2024-11-27

**Authors:** Amine Ben-Daoued, Frédéric Bernardin, Pierre Duthon

**Affiliations:** Cerema, Research Team “Intelligent Transport Systems”, 8-10 Rue Bernard Palissy, CEDEX 2, F-63017 Clermont-Ferrand, France; frederic.bernardin@cerema.fr (F.B.); pierre.duthon@cerema.fr (P.D.)

**Keywords:** fog, radiative transfer, automotive sensors

## Abstract

Accurate luminance-based image generation is critical in physically based simulations, as even minor inaccuracies in radiative transfer calculations can introduce noise or artifacts, adversely affecting image quality. The radiative transfer simulator, SWEET, uses a backward Monte Carlo approach, and its performance is analyzed alongside other simulators to assess how Monte Carlo-induced biases vary with parameters like optical thickness and medium anisotropy. This work details the advancements made to SWEET since the previous publication, with a specific focus on a more comprehensive comparison with other simulators such as Mitsuba. The core objective is to evaluate the precision of SWEET by comparing radiometric quantities like luminance, which serves as a method for validating the simulator. This analysis is particularly important in contexts such as automotive camera imaging, where accurate scene representation is crucial to reducing noise and ensuring the reliability of image-based systems in autonomous driving. By focusing on detailed radiometric comparisons, this study underscores SWEET’s ability to minimize noise, thus providing high-quality imaging for advanced applications.

## 1. Introduction

The automotive industry is constantly evolving and developing new technologies to improve the safety of drivers and vulnerable road users. In particular, Advanced Driver Assistance Systems (ADAS) use sensors, such as lidars, radars, and cameras, to detect and identify objects in the environment to avoid collisions. To ensure that these sensors are accurate and reliable, it is necessary to evaluate them in exhaustive and critical situations. However, the evaluation of these sensors is a complex and costly process, as it requires extensive real-life testing. Numerical simulation offers a viable alternative to this process, as it allows for the massive testing of sensors in a cost-effective and safe manner. This is where SWEET (Simulating WEather for intElligent Transportation systems [1]), a physically-based simulator developed by Cerema, comes in.

The choice to develop our own simulator, SWEET, from scratch was driven by the need for independence from existing simulator implementations, which can sometimes limit customization when integrating new simulation components tailored to our specific research objectives. SWEET was designed to meet both the internal research requirements of our team and the unique needs of the fog and rain PAVIN platform. By developing and mastering each component of SWEET, we ensure that every aspect of the simulator aligns precisely with our needs. This approach also allows us to thoroughly verify each simulation module by comparing its outputs with experimental measurements obtained from our PAVIN platform, thereby enhancing the accuracy and reliability of our work.

The development of SWEET is driven by a primary goal: to deeply understand the physics underlying the equations, to thoroughly master the processes involved, and to verify each component of this simulator through analytical means (when it is possible) and to validate it with real measurements. These measurements may come from natural data or data acquired within our fog and rain PAVIN platform [2,3]. Our overarching objective was to create a simulator that is both open for reasearch topics and industrialized, serving as a reference point for various applications in the evaluation of autonomous vehicle sensors. For example, it can be used to test and validate the performance of autonomous vehicle sensors before they are deployed in the field. This can help to ensure that these sensors are reliable and accurate, which is crucial for the safety of autonomous vehicles. In this context, the SWEET simulator represents an extension to the range of services offered by Cerema with its PAVIN fog and rain platform.

In the domain of road perception simulators, two distinct families have emerged to address the challenges posed by degraded weather conditions, particularly fog. The first family comprises physically-based simulators, exemplified by SWEET ([1]) and Mitsuba ([4]), which model the interaction of light with the environment using physical full path tracing methods combined with a Monte Carlo (MC) scheme imitating the movement of photons in space. The second family consists of simplified simulators, such as Carla [5,6], 4DVirtualiz [7], LGSLV [8], CarSim [9], PreScan [10], Airsim [11], PTV Vissim [12], AVSimulation SCANeR [13], IPG CarMaker [14] and Pro-SiVIC [15], which rely on simplified physical laws such as the Beer-Lambert law or statistical data by training perception algorithms on real or simulated databases. The latter family has gained popularity in the autonomous vehicle industry due to their real-time capabilities, making them suitable for integration into sensor evaluation loops requiring real-time feedback (e.g., X-in-the-loop tests). On the other hand, physically-based simulators offer a high-fidelity representation of the environment, accounting for complex interactions between light and matter. These simulators are capable of accurately modeling the effects of fog on visibility and sensor performance. However, their computational demands can be significant, limiting their real-time applicability. However, simplified simulators, such as Carla, provide a computationally efficient alternative. They often rely on simplified physical models or statistical data to approximate the effects of fog on visibility and sensor performance. While these simulators may lack the accuracy and fidelity of physically-based approaches, they offer the advantage of real-time performance, making them suitable for rapid prototyping and testing in the automotive industry. In our last article [1], we already compared renderings of road scenes from SWEET with those produced by Mitsuba. In that work, we focused on evaluating the relevance of the results obtained using SWEET through low-level metrics based on contrast analysis, specifically comparing its outputs with those from Mitsuba and real images. While other comparison methods can utilize high-level metrics based on object detection algorithms [16], the present paper advances the discussion further by conducting comparisons on radiometric quantities, including luminance and fluence. By doing so, it provides deeper insights into the performance of SWEET in simulating realistic lighting interactions, thereby enhancing the understanding of its capabilities and limitations in practical applications. We focus on the first family of simulators cited before, specifically comparing the performance of SWEET and Mitsuba in weather-affected road perception scenarios. We aim to evaluate the accuracy and computational efficiency of these simulators in modeling fog and its impact on sensor performance. By conducting a comparative study, we seek to provide insights into the strengths and limitations of physically-based simulators in addressing the challenges of degraded weather conditions in automotive applications. Indeed, among the strongest handicaps of today’s MC algorithms, are the difficulties related to numerical behavior in the optically thick limit ([17,18,19]), which can introduce significant biases in perception applications. Ref. [20] mentions that MC algorithms, without any biasing mechanisms, start to break down for transverse optical depths τ≥ 20. Despite these challenges, MC simulators remain valuable tools for simulating light transport in complex media and understanding the underlying physical processes. Their ability to handle heterogeneous and highly scattering environments makes them indispensable for various applications, including atmospheric science, biomedical imaging, and computer graphics. However, it’s essential to knowledge their limitations and explore alternative approaches, such as hybrid methods or optimization techniques, to address scenarios where traditional MC simulations falter. We aim to shed light on these issues, provide insights into the applicability of MC methods in road perception scenarios and summarize the improvments that can be done to reduce the variance.

Monte Carlo codes, like any other codes that solve integro-differential equations, must undergo rigorous verification and validation procedures [21,22,23]. Verification refers to a series of procedures to ensure that a code is computationally sound, meaning it performs the calculations it is intended to without any bugs [21,22,23]. Validation, on the other hand, involves comparing the numerical results generated by the code with experimental results from a physical system that the code is designed to model [22,24].

The existing literature identifies three primary procedures for verifying MC codes:

- Comparison with previously verified MC codes [25,26,27,28,29,30,31,32,33]: This method relies on the use of established MC codes that have already been verified. However, this approach is limited by the finite accuracy of the MC data generated. Small discrepancies in the data can lead to significant uncertainties in the verification process. This verification step is the subject of this paper, the two other steps below will be presented in future in a seperate paper.
- Comparison with solutions of the radiative transfer equation (RTE): In tissue optics, benchmark RTE solutions, often provided by researchers such as van de Hulst [34] and Giovanelli [26,28,35,36], are used as references. These solutions are mostly in semi-analytical form and are available for regularly bounded geometries, such as semi-infinite media or layers with uniform scattering properties. Despite their utility, this verification method has two major drawbacks: the solutions cannot typically be expressed in closed form and are known with limited accuracy. Furthermore, they may be affected by convergence issues, leading to potential inaccuracies in the calculated quantities.
- Comparison with asymptotic solutions of the diffusion equation [27,29,37]: While the diffusion equation offers asymptotic solutions that are used in the literature, this approach is constrained by the inherent approximations of diffusion theory. These solutions cannot be applied universally across all scenarios covered by the RTE. The accuracy of diffusion equation solutions is further compromised in finite geometries or in cases involving scattering and/or refractive index mismatches between different regions of the medium.

Another lesser-known method for verifying MC methods is based on the invariance property (IP) [21,38,39,40,41]. The core of this method lies in the well-known, but not widely utilized, invariance property of the mean path length traveled by a random walker within a disordered medium. This property holds when the entrance of particles into the medium occurs homogeneously and isotropically, and no particle absorption or annihilation takes place within the medium [39,42,43]. In optical contexts, this corresponds to a constant incoming radiance, often referred to as Lambertian illumination. Under these conditions, the result is a constant mean path length that depends solely on the basic geometric characteristics of the medium, regardless of the distribution of scattering properties.

The IP verification method can effectively test the correctness of MC codes in more complex geometries and distributions of optical properties. It is capable of detecting various types of errors in an MC code and is particularly sensitive to inaccuracies in the treatment of boundaries. Common errors include incorrect modeling of light propagation (including interactions with boundaries) and implementation errors within the code, such as systematic inaccuracies due to the finite precision of built-in functions [25,32].

This verification procedure offers a powerful and universal tool for ensuring the accuracy of MC simulations. By leveraging the invariance property, which is inherently robust against variations in scattering properties and specific boundary conditions, this method provides an additional layer of validation that complements traditional methods.

This paper makes several key contributions to the field of validation and verification of virtual testing methods for autonomous vehicle sensor evaluation. First, it highlights the advancements of the SWEET simulator, which is valuable for both our team and the broader autonomous vehicle community, particularly in simulating complex weather scenarios with high fidelity. By introducing a methodology based on radiometric comparisons (luminance, fluence), this work establishes a new approach for assessing the robustness of Monte Carlo calculations, especially in applications where physically-based image generation relies on these quantities. Additionally, the study brings attention to the limitations of Monte Carlo schemes, specifically the backward scheme employed in SWEET, and identifies possible improvements. Finally, these findings are contextualized with respect to applications for autonomous vehicle perception sensors, such as cameras, emphasizing the practical implications of accurate light modeling for sensor performance in degraded weather conditions.

In this paper, we firstly detail in Section 2 the context of the automotive industry as well as the needs in the context of autonomous vehicle applications. This will make it possible to identify the ranges of interest for each of the simulation parameters to be taken into account for the verification. Then in Section 3, we address the theoretical foundations and assumptions of the Monte Carlo methods that solve the radiative transfer equation and we show the new features in the implementation of SWEET compared to its latest version used in our last paper [1]. In Section 4.1, we compare SWEET to other Monte Carlo-based codes, both Mitsuba and Steven’s code [44], which evaluates fluence in participating media, particularly tissues in medical contexts. We also check the invariance property of SWEET in section Section 4.2 and a computational time analysis is done in Section 4.3 before concluding.

## 2. General Need for the Automotive Industry

As explained in Section 1, the SWEET simulator has been developed to meet the needs of ADAS and Automated Driving System (ADS) validation and certification. To achieve this, the simulator must be as representative of reality as possible, with particular attention paid to the following elements: 3D scene geometry, surface materials, lighting, sensors, vehicle dynamics, weather conditions, etc. The SWEET simulator is currently being tested and validated on these different aspects within the ROADVIEW project [45]. For the purposes of this article, we restrict ourselves to verifying the simulator’s performance in a fog environment, using a light source and a simple scene.

To achieve this, it is important to validate the behavior of the fog generated in the SWEET simulator within its proper range of use, specifically in the automotive context. The fog parameters for which a verification range must be determined are as follows:

- Optical thickness (corresponding to the combination of fog density and observation distance).
- The ratio between the light sources and the sensor sensitivity, which also defines an equivalent optical thickness limit.

To define the range of optical thickness to be considered for the simulator, the maximum expected detection distance must be established alongside the meteorological optical range (MOR) of the fog present in the road scene. The meteorological visibility, or meteorological optical range (MOR), is defined as the distance at which the luminous flux of a collimated light beam at 550 nm is reduced to 5% of its initial value [46,47]. According to this definition, the MOR is related to the extinction coefficient β as follows:

(1) $$MOR=\frac{-\ln(0.05)}{\beta }\approx \frac{3}{\beta },$$

First of all, current vehicle perception systems have maximum detection distances of 100 m on a clear day [48]. In a practical case, this means that obstacles can be detected with certainty 3 s before impact, at a speed of 130 km/h.

Regarding the fog, standard NF-P99-320 [49] defines the presence of fog in a road context when the MOR is below 400 m. In nature, there are occurrences of extremely dense fog with an MOR of 10 m [50]. However, for an MOR below 50 m, the French highway code requires vehicle speed to be reduced to 50 km/h.

Using this data, it is possible to determine the maximum optical thickness that the simulator should be able to simulate, covering cases from light fog to extremely dense fog. As shown in Table 1, the densest fog corresponds to the highest optical thickness, at various speed limits. Therefore, this is the most restrictive case, with an optical thickness of 12.5. Table 1 also presents another method for calculating the maximum optical thickness: the maximum optical thickness that can be physically generated in the PAVIN fog and rain platform. This limit is relevant because the next phase will be to validate the SWEET simulator using real data collected from this platform.

As shown in Table 1, the platform can reproduce an optical thickness of up to 18.8, so the simulator will need to be able to simulate fog with an optical thickness of around 20 to account for all the real-life situations it may encounter. The verification of the SWEET simulator in this article will thus be conducted for optical thicknesses ranging from 0 to 20.

Another way of determining an equivalent optical thickness limit to simulate is to establish the ratio between the light intensity of the source and the sensitivity of the sensors. In the verification simulations carried out in this article, we consider a source of intensity 1 and analyze the results of different simulation methods. It is therefore important to define a low threshold for the receiver, below which the results are not relevant in the automotive context.

Regarding the ratio between the light intensity of the sources and the sensitivity of the sensors, several scenarios can be considered. For this, limit cases must be identified. These cases are derived by considering the most powerful source possible and the minimum detection value of the sensor. We provide a summary of these cases in Table 2, which we will detail later.

Firstly, we can consider a car camera in daylight, with a scene illuminated by sunlight. In this scenario, the minimum irradiance value that a camera can detect is determined based on the standard characteristics of automotive cameras. This threshold corresponds to the point where it is no longer possible to distinguish signal from noise. We consider the irradiance of the sun and assume that it illuminates a Lambertian surface with a reflectivity of 1. By doing this, we obtain the radiance in the direction of the camera, resulting in an optical thickness of 16.1, as shown in Table 2.

Next, we consider the case of vehicle headlights in night conditions, facing an automotive camera. For the camera, we use the same assumptions as in daylight conditions. For the headlamp, we assume that it is collimated, a simplifying assumption that will tend to increase the limiting optical thickness and thus improve safety. By considering the power of the headlamp, we can again determine an optical thickness, which is 22.4 in this second example, as shown in Table 2.

Finally, similar to the fog scenario, we estimate the optical thickness by considering the limits of our equipment, which will be used for the second validation phase. In this phase, we will use a light source and a spectroradiometer, with their characteristics listed in Table 2. Using a similar approach as for the other examples, we can determine the maximum measurable optical thickness for this setup, which is 10.4.

In conclusion, this new analysis shows that simulating a maximum optical thickness of 20 (defined through the analysis of fog limit cases in a road context) is also sufficient to meet the limitations imposed by vehicle light sources and sensors. In the remainder of this article, we will only consider scenarios with a source of a unit intensity and a maximum optical thickness of 20 for simulation.

Thanks to an analysis of different scenarios in the automotive context (with testing ADS and ADAS as the ultimate objective of the SWEET simulator), we were able to determine the simulation limits to be considered in the rest of our study: an optical thickness range of [0–20]. We now present our verification methods in the next section.

## 3. Models and Implementation

### 3.1. The Radiative Transfer Equation

The radiative transfer equation (RTE) serves to simulate the radiance $L_{\lambda }\left(t,r,u\right)$ corresponding, for a wavelength λ, to the intensity of the electromagnetic energy flux (in W) of the radiation propagating in the direction *u*, per unit of area (in m^2^) perpendicular to the direction of propagation, per unit of solid angle (in sr) and per unit of wavelength (in microns), and expressed in W/m^2^/μ/sr.

We recall here the RTE [51]:

(2) $$\begin{array}{c} \frac{1}{c}\frac{\partial L_{\lambda }}{\partial t}\left(t,r,u\right)+u\cdot \nabla_{r}L_{\lambda }\left(t,r,u\right)=-\sigma_{\lambda }\left(r\right)L_{\lambda }\left(t,r,u\right)-\kappa_{\lambda }\left(r\right)L_{\lambda }\left(t,r,u\right)\\ +\frac{\sigma_{\lambda }\left(r\right)}{4\pi }\int_{S^{2}}L_{\lambda }\left(t,r,v\right)\Phi_{\lambda }\left(r,v,u\right)dv+q(t,r,u), \end{array}$$

where *c* is the speed of light, *t*, *r*, *u*, $\sigma_{\lambda }$, $\kappa_{\lambda }$, $\Phi_{\lambda }$ and q(t,x,u) denote, respectively, the time, the position in space, the wave propagation direction, the scattering coefficient, the absorption coefficient, the phase function for the wavelength λ and a space continuous source. The three-dimensional unit sphere is denoted by $S^{2}$. By using the vocabulary dedicated to the rendering volumetric path tracing method, the terms in blue and green represent the out-scattering and absorption terms, respectively. These two terms together form the extinction term. The term in red corresponds to the in-scattering term, which is the most expensive part to calculate as it requires integration over all paths in the scene. Finally, the term in brown is for emission. Optical passive objects and local light sources are taken into account thanks to the boundary conditions of Equation (2). For each light source occupying the space region *S* and emitting light from its surface ∂S, we have:

(3) $$\forall \;r\in \partial S,\;\forall \;u\in S^{2},\;u\cdot n_{r}^{S}>0,\;L_{\lambda }\left(t,r,u\right)=E_{\lambda }^{S}\left(t,r,u\right),$$

where $n_{r}^{S}$ denotes the outward normal vector of *S* at point r∈∂S, and $E_{\lambda }$ is given. For each passive object occupying the space region *O* with a surface denoted by ∂O, we have:

(4) $$\forall \;r\in \partial O,\;\forall \;u\in S^{2},\;u\cdot n_{r}^{O}>0,\;L_{\lambda }\left(t,r,u\right)=\int_{v\in S^{2},\;v\cdot n_{r}^{O}<0}L_{\lambda }\left(t,r,v\right)B_{\lambda }^{O}\left(r,v,u\right)dv,$$

where $n_{r}^{O}$ denotes the outward normal vector of *O* at point *r*, and $B_{\lambda }^{O}\left(r,\cdot ,\cdot \right)$ is the Bidirectional Reflectance Distribution Function (BRDF) of object *O* at point r∈∂O. When the time can be removed from the physics and under the assumption of a phase function depending only on v·u (scalar product), the following stationary case of Equation (2) can be considered:

(5) $$u\cdot \nabla_{r}L_{\lambda }\left(r,u\right)=-\beta_{\lambda }L_{\lambda }\left(r,u\right)+\frac{\sigma_{\lambda }}{4\pi }\int_{S^{2}}L_{\lambda }\left(r,v\right)\Phi_{\lambda }\left(v\cdot u\right)dv+q\left(r,u\right),$$

where we note $\beta_{\lambda }=\sigma_{\lambda }+\kappa_{\lambda }$ the extinction coefficient at the wavelength λ. Boundary conditions related to this stationary case are given by Equations (3) and (4) in which the time *t* is removed.

### 3.2. Implementation of SWEET

In our previous article, we detailed the implementation specifics of SWEET ([1]), a full path-tracing-based simulator designed to evaluate interactions with a participating medium, considering a tabulated drop size distribution (DSD). This implementation was parallelized on the GPU and utilized the Surface Area Heuristic (SAH) kd-Tree acceleration technique and was limited to only diffuse material surfaces. In this article, we present an updated version of SWEET that incorporates several enhancements, including improved variance reduction techniques, importance sampling on lights and materials, and the inclusion of Bidirectional Scattering Distribution Functions (BSDFs), the Henyey-Greenstein phase function for the light scattering property of the fog and several analytical light models. Next subsections detail the main enhancements of SWEET.

#### 3.2.1. Interaction of Light with Materials

The BRDF is a fundamental concept in physically based rendering and optical simulations. It describes how light is reflected at an opaque surface. Specifically, the BRDF defines the relationship between incoming light (radiance) and the reflected light at a point on a surface, depending on the direction of both the incident and outgoing light [52].

Mathematically, the BRDF is defined as the ratio of the radiance reflected in the outgoing direction to the irradiance incident from a specific incoming direction [52]. The BRDF is a function of four variables: the incoming direction $\omega_{i}$, the outgoing direction $\omega_{o}$, and the surface normal *n* (two angles for $\omega_{i}$ and two angles for $\omega_{o}$, the surface normal *n* is implicitely determined at a given point of the surface). It is typically written as $f_{r}\left(\omega_{o},\omega_{i}\right)$, where $\omega_{o}$ and $\omega_{i}$ are unit vectors representing the outgoing and incoming directions, respectively [53].

The BRDF, $f_{r}\left(\omega_{o},\omega_{i}\right)$, is defined as:

(6) $$f_{r}\left(\omega_{o},\omega_{i}\right)=\frac{dL_{o}\left(\omega_{o}\right)}{dE_{i}\left(\omega_{i}\right)}$$

where:

- $f_{r}\left(\omega_{o},\omega_{i}\right)$ is the BRDF, which gives the ratio of the reflected radiance to the incoming irradiance.
- $dL_{o}\left(\omega_{o}\right)$ is the differential outgoing radiance in the direction $\omega_{o}$.
- $dE_{i}\left(\omega_{i}\right)$ is the differential irradiance coming from direction $\omega_{i}$.

The outgoing radiance $L_{o}\left(\omega_{o}\right)$ from a surface point in the direction $\omega_{o}$ can be computed by integrating the incoming radiance $L_{i}\left(\omega_{i}\right)$ from all directions over the hemisphere Ω above the surface [53]. The BRDF $f_{r}\left(\omega_{o},\omega_{i}\right)$ modulates how much of the incoming radiance is reflected in the outgoing direction.

The outgoing radiance $L_{o}\left(\omega_{o}\right)$ is given by the following integral:

(7) $$L_{o}\left(\omega_{o}\right)=\int_{\Omega }f_{r}\left(\omega_{o},\omega_{i}\right)L_{i}\left(\omega_{i}\right)\left(\omega_{i}\cdot n\right)\;d\omega_{i}$$

where:

- $L_{o}\left(\omega_{o}\right)$ is the outgoing radiance in the direction $\omega_{o}$.
- $L_{i}\left(\omega_{i}\right)$ is the incoming radiance from direction $\omega_{i}$.
- $f_{r}\left(\omega_{o},\omega_{i}\right)$ is the BRDF, which determines the fraction of the incoming radiance reflected in the outgoing direction.
- $\left(\omega_{i}\cdot n\right)$ is the cosine of the angle between the incoming direction $\omega_{i}$ and the surface normal *n*. This accounts for the foreshortening effect (i.e., the surface receives less irradiance as the angle of incidence increases).
- $d\omega_{i}$ is the differential solid angle in the direction of the incoming light.
- Ω represents the hemisphere above the surface, i.e., the domain of all possible incoming light directions.

In many modern simulators, a more general model called the BSDF is used. The BSDF combines both the BRDF, which describes reflection, and the Bidirectional Transmission Distribution Function (BTDF), which describes transmission (light passing through a surface) [54]. The BSDF therefore covers both reflective and transmissive behaviors of light interacting with surfaces.

In more comprehensive models like the BSDF, both reflection and transmission are considered [53]. The BSDF, $f_{s}\left(\omega_{o},\omega_{i}\right)$, unifies the BRDF and BTDF (Bidirectional Transmission Distribution Function). For reflective interactions, the BRDF describes how light is reflected, while for transmissive interactions, the BTDF describes how light is transmitted through the material [54].

The BSDF is given by:

(8) $$f_{s}\left(\omega_{o},\omega_{i}\right)=f_{r}\left(\omega_{o},\omega_{i}\right)+f_{t}\left(\omega_{o},\omega_{i}\right)$$

where:

- $f_{s}\left(\omega_{o},\omega_{i}\right)$ is the BSDF, representing both reflection and transmission.
- $f_{r}\left(\omega_{o},\omega_{i}\right)$ is the BRDF, handling the reflected light.
- $f_{t}\left(\omega_{o},\omega_{i}\right)$ is the BTDF, handling the transmitted light.

**Figure 1:**
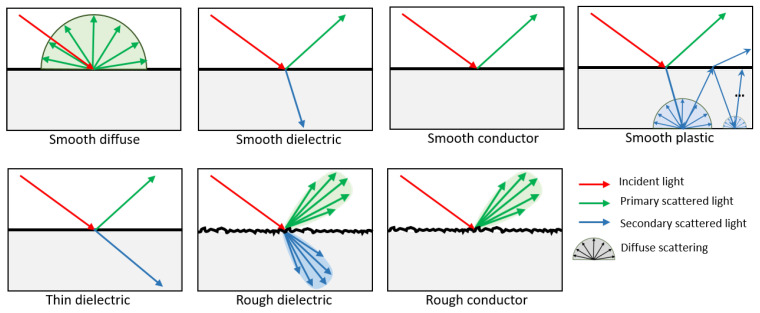
Schematic overview of the most important surface scattering models in SWEET.

In the SWEET simulation environment, several BSDF models are integrated to simulate how light interacts with various materials. These BSDF models (shown in Figure 1) are crucial for accurately depicting the behavior of light as it reflects, transmits, and scatters across surfaces, including anisotropic surfaces, which are commonly found in real-world environments. The physically based models allow the rendering system to adhere to the real-world physics of light interaction, which is particularly important for applications in optical simulations, such as camera imaging in automated vehicles. Below is a detailed explanation of the different BSDF models available in SWEET and their characteristics.

#### Diffuse (Lambertian)

A diffuse BSDF, commonly referred to as Lambertian, assumes that light is scattered uniformly in all directions from a surface. It models perfectly matte surfaces where the reflection does not depend on the viewer’s angle. The outgoing radiance is proportional to the cosine of the angle between the incident light and the surface normal, making it angle-independent.

- **Example**: White paint, chalk, or matte surfaces like plaster walls.
- **Microfacet Model**: N/A (no microfacets are involved in pure diffuse reflection).

#### Smooth Dielectric

Smooth dielectric BSDFs model transparent or translucent materials where light passes through the material, reflecting and refracting at smooth surfaces. Dielectrics are materials that do not conduct electricity and have minimal absorption of light. Smooth surfaces imply that there is little to no surface roughness, allowing for sharp, specular reflections and transmissions governed by Fresnel equations.

- **Example**: Glass, water, or clear plastic.
- **Microfacet Model**: N/A (smooth surfaces do not require a microfacet model).

#### Thin Dielectric

The thin dielectric model simulates very thin layers of dielectric materials, such as coatings or films, where interference effects can play a role. This model is crucial for capturing subtle effects like color changes due to interference, seen in thin films like soap bubbles or anti-reflective coatings.

- **Example**: Soap bubbles, anti-glare coatings on lenses.
- **Microfacet Model**: N/A (often treated without microfacets due to thinness).

#### Rough Dielectric

A rough dielectric BSDF handles materials that are similar to smooth dielectrics but with surface roughness. Roughness introduces a more complex scattering behavior, where light is reflected and refracted in a more diffuse manner. The scattering is modeled using microfacet theory, where the surface is composed of tiny facets that each reflect light based on their orientation.

- **Example**: Frosted glass, rough plastic, or sandblasted glass.
- **Microfacet Model**: Beckmann, GGX [55].

#### Smooth Conductor

Smooth conductor BSDFs are used to simulate metals and other conductive materials, where light is primarily reflected rather than transmitted. These materials exhibit high reflectivity and may produce colorful reflections based on the wavelength-dependent refractive index of the material (complex index of refraction (IOR)). Since the surface is smooth, the reflections are sharp and mirror-like.

- **Example**: Polished copper, gold, or aluminum.
- **Microfacet Model**: N/A (smooth surfaces reflect light in a specular manner).

#### Rough Conductor

Rough conductor BSDFs simulate the behavior of light on rough metal surfaces. As with rough dielectrics, rough conductors scatter light due to surface microgeometry, which leads to blurred or diffuse reflections. The interaction is modeled using microfacet theory, where the surface consists of many tiny facets, each reflecting light at different angles depending on their orientation.

- **Example**: Brushed metal, oxidized or textured metals.
- **Microfacet Model**: Beckmann, GGX [55]

#### Plastic

The plastic BSDF combines characteristics of both dielectric and diffuse materials. Plastics generally have a diffuse base layer with a glossy, specular coating on top. This means light interacts with the material in two ways: part of the light is diffusely reflected, and part is reflected specularly depending on the smoothness of the surface.

- **Example**: Car paint, polished plastic surfaces.
- **Microfacet Model**: current version only supports smooth plastic (no microfacet model needed).

A summary of these BSDF models implemented in SWEET can be found in Table 3.

In other simulators, we may encounter what is called a “Principled BSDF”. This type of BSDF is designed to simplify the process of shading and material creation by offering a more artist-friendly approach. It combines several different shading models, such as diffuse, glossy, and subsurface scattering, into one unified shader. The Principled BSDF is often used in rendering engines aimed at generating aesthetically pleasing images, like those for movies, games, or other creative industries. While the Principled BSDF is excellent for producing visually appealing results quickly, it is not always appropriate for physically based applications, especially in scientific simulations like those performed in SWEET. In these contexts, accuracy in the light-matter interaction is critical for reliable simulation results, as we focus on exact calculations of physical quantities like luminance and fluence. SWEET requires detailed BSDF models, such as those for rough dielectrics and conductors, to accurately replicate real-world physics, making it better suited for tasks like radiative transfer and optical simulations, particularly in demanding fields like automated vehicle imaging.

The integration of a tabulated (measured) BRDFs in a path tracing simulator such as SWEET can be computationally expensive, that’s why empirical models are often used in the literature. However, we plan to explore tabulated BRDFs in a future version of SWEET, leveraging acceleration techniques to mitigate computational costs. Additionally, we intend to implement textures, which can enhance the realism of materials by adding visual details such as patterns or imperfections. These details will improve rendering quality and precision while optimizing computational efficiency. Ongoing work with the gonioreflectometer at Cerema focuses on measuring the BRDFs of various material samples. These measured BRDFs will subsequently be compared to the results from the SWEET simulator to calibrate the implemented BRDF models. This research is anticipated to lead to a future publication, contributing valuable insights into the accuracy and reliability of reflectance modeling in simulation environments.

#### 3.2.2. Interaction of Light with Participating Media

The inclusion of the Henyey-Greenstein phase function (Equation (9)) allows for the simulation of anisotropic scattering in a medium, which is essential for accurately modeling the behavior of light in foggy or hazy environments. This feature enhances the realism of the simulations and enables more accurate predictions of visibility and sensor performance in adverse weather conditions. This phase function is advantageous because it bypasses the uncertainties that may exist in tabulated phase functions (as these tabulated phase functions are calculated considering a measured DSD). This can be useful to overcome this step and validate models without having to worry about measurement uncertainties. The Henyey-Greenstein phase function is widely used in various fields, including atmospheric science ([56]), remote sensing, and biomedical optics ([57,58,59,60,61,62]). This phase function is particularly useful in optical simulations, as it provides a good approximation for the scattering behavior of various types of media. It is defined by a single parameter, the asymmetry parameter, which controls the directionality of the scattering. This parameter can be adjusted to simulate different types of scattering, from forward scattering (where light is predominantly scattered in the forward direction) to isotropic scattering (where light is scattered in all directions).

(9) $$P\left(\cos\theta \right)=\frac{1-g^{2}}{{\left(1+g^{2}-2g\cos\theta \right)}^{3/2}}\;\mathrm{for}\;-1\leq g\leq 1$$

where:

- P(cosθ) = Phase function, which represents the probability of scattering at an angle θ.
- θ = Scattering angle, which is the angle between the incident light direction and the scattered light direction.
- *g* = Asymmetry parameter, which describes the average cosine of the scattering angle. It ranges from −1 to 1, where:
  – g<0 indicates backward scattering,
  – g=0 indicates isotropic scattering, and
  – g>0 indicates forward scattering.

#### 3.2.3. Variance Reduction Techniques

The improved variance reduction techniques, such as importance sampling on lights and materials (BSDFs), help to reduce noise and improve the convergence in volumetric path tracing, especially in complex lighting scenarios. This results in more accurate and reliable results, especially in scenes with complex lighting and materials. These techniques are popular in the literature ([63,64,65,66,67]. However, each of these two importance sampling strategies has a high variance in some situations. For example, if the light source is small and the material is quite diffuse, then importance sampling of the material can result in high variances. Conversely, if the source is large and the material is shiny, importance sampling of the light source can lead to high variances. Neither of these strategies is valid across the entire integration domain of the reflection equation (Equation (7)).

The use of both strategies simultaneously (by combining them), as implemented in SWEET, results in what is called multiple importance sampling (MIS) and is an advantageous solution ([63,67]). Chapter 9 of Veach’s thesis ([63]) is a particularly valuable reference that details these strategies as well as MIS. Indeed, MIS is a powerful technique used in rendering to reduce variance when combining different sampling strategies, particularly when sampling from light sources and BSDFs [63]. It is particularly useful when there are multiple light sources or when the material properties vary significantly across the scene. MIS works by assigning a weight to each sample based on its probability of being selected by each sampling strategy. These weights are then used to combine the samples into a single estimate of the integral. The balance heuristic is a commonly used weighting method that helps in effectively combining these strategies [63]. MIS can significantly reduces variance compared to using a single sampling strategy, especially in scenes with complex lighting or materials. However, it requires careful tuning of the sampling probabilities to ensure that the weights are balanced and that the resulting estimate is unbiased.

The outgoing radiance *L* can be estimated by combining contributions from light samples $L_{e}$ and BSDF samples $L_{b}$:

(10) $$L\left(x,w\right)=\sum_{i=1}^{N_{L}}L_{e}\left(x,w_{i}\right)\cdot p_{L}\left(w_{i}\right)+\sum_{j=1}^{N_{B}}L_{b}\left(x,w_{j}\right)\cdot p_{B}\left(w_{j}\right)$$

where:

- L(x,w) = Total outgoing radiance at point x in direction w.
- $L_{e}\left(x,w_{i}\right)$ = Emission from the *i*-th light sample.
- $L_{b}\left(x,w_{j}\right)$ = Contribution from the *j*-th BSDF sample.
- $p_{L}\left(w_{i}\right)$ = Probability density function (PDF) of the *i*-th light sampling direction.
- $p_{B}\left(w_{j}\right)$ = Probability density function (PDF) of the *j*-th BSDF sampling direction.
- $N_{L}$ = Total number of light samples.
- $N_{B}$ = Total number of BSDF samples.

The estimate can be improved by using the balance heuristic for weighting the contributions from light and BSDF samples:

(11) $$L\left(x,w\right)=\frac{1}{N}\left(\sum_{i=1}^{N_{L}}L_{e}\left(x,w_{i}\right)\cdot \frac{w_{L}\left(w_{i}\right)}{p_{L}\left(w_{i}\right)}+\sum_{j=1}^{N_{B}}L_{b}\left(x,w_{j}\right)\cdot \frac{w_{B}\left(w_{j}\right)}{p_{B}\left(w_{j}\right)}\right)$$

where:

- *N* = Total number of samples (both light and BSDF).
- $w_{L}\left(w_{i}\right)$ = Weight for the *i*-th light sample.
- $w_{B}\left(w_{j}\right)$ = Weight for the *j*-th BSDF sample.

The weights can be calculated using the balance heuristic as follows:

(12) $$w_{L}\left(w\right)=\frac{p_{L}\left(w\right)}{p_{L}\left(w\right)+p_{B}\left(w\right)},\;w_{B}\left(w\right)=\frac{p_{B}\left(w\right)}{p_{L}\left(w\right)+p_{B}\left(w\right)}$$

where:

- $w_{L}\left(w\right)$ and $w_{B}\left(w\right)$ ensure that the contributions are properly normalized and weighted according to the sampling strategies used.

In addition to the variance reduction techniques mentioned earlier, we have also integrated a termination mechanism, a Russian roulette feature ([68]), into SWEET. The user has the option to disable this feature if necessary. Russian roulette is a technique used in Monte Carlo simulations to reduce computational time by randomly terminating paths that have a low contribution to the final result. This is achieved by assigning a probability of termination to each path, based on its contribution to the overall result. Paths that are terminated are replaced by a weighted average of the paths that continue, ensuring that the overall result remains unbiased. Overall, the Russian roulette feature in SWEET provides a flexible and efficient way to reduce computational time in Monte Carlo simulations, without compromising the accuracy of the results.

The models and implementation details of the SWEET simulator have just been presented. In the following sections, we present the verification results of SWEET using the methods mentioned in the introduction (comparisons with other MC-based simulators in the literature, as well as the results of the invariance property test). For all simulations, we maximized the computational resources available to us. For SWEET, the calculations were performed on two separate Linux machines: the first equipped with an NVIDIA GeForce GTX 1080 graphics card, with a maximum Monte Carlo iteration *N* up to $10^{8}$, and the second equipped with an NVIDIA RTX A4500 graphics card, with a maximum Monte Carlo iteration count *N* up to $1.2\times 10^{8}$. These high iteration counts enabled us to push the accuracy of our simulations to the limits of our hardware capabilities, ensuring robust and reliable verification results.

## 4. Results

### 4.1. Comparing SWEET to Other Monte Carlo-Based Codes

The comparison with other Monte Carlo simulators serves initially to position SWEET among these simulators, offering a valuable theoretical verification of its capabilities. However, it’s essential to recognize that all Monte Carlo simulators have inherent limitations, particularly when it comes to handling certain scenarios, such as thick optical depths ([17,18,19,20]). These limitations arise primarily from computational constraints and the fundamental assumptions underlying Monte Carlo methods.

One of the primary challenges faced by Monte Carlo simulators is their computational complexity, which increases exponentially with the optical thickness of the medium being simulated. As the optical depth increases, the number of scattering events and interactions that need to be simulated also grows, leading to significant computational overhead. Beyond a certain threshold, Monte Carlo simulations become computationally infeasible or prohibitively time-consuming, limiting their applicability in scenarios with high optical thicknesses, such as dense fog or thick clouds. Furthermore, Monte Carlo simulations rely on stochastic sampling techniques to approximate solutions, introducing inherent statistical uncertainties. While this probabilistic approach is well-suited for modeling complex and random processes, it can also result in variance and convergence issues, particularly in scenarios with low photon counts or rare events. Consequently, Monte Carlo simulators may struggle to provide accurate and reliable predictions in extreme or outlier situations, further highlighting their limitations.

We emphasize that our choice to compare SWEET with Mitsuba was deliberate, as Mitsuba is well-known in the realm of physics-based simulators. Furthermore, this decision was influenced by our prior work where we compared the visual rendering capabilities of SWEET with those of Mitsuba. By extending this comparison to include aspects beyond visual rendering, we aim to provide a comprehensive evaluation of SWEET’s performance against a widely recognized benchmark in the field. Similarly, the decision to compare SWEET with Steven’s code was motivated by several factors. Steven’s code represents another physics-based simulator, which aligns well with the objectives of our study. Specifically, Steven’s code addresses the scenario of a point source in an infinite medium, mirroring the context of our comparison scenario. Additionally, the availability of Steven’s code online and its ease of access make it an attractive choice for comparison purposes, facilitating reproducibility and transparency in our evaluation process. Moreover, comparing SWEET with Steven’s code allows us to contrast the Monte Carlo forward scheme utilized in Steven’s code with the backward scheme employed in SWEET. This comparison sheds light on the strengths and weaknesses of each approach, providing valuable insights into the underlying methodologies and their implications for simulation accuracy and efficiency. By juxtaposing these different Monte Carlo schemes, we can elucidate the relative merits of each approach and identify opportunities for optimization and refinement. Overall, the strategic selection of Mitsuba and Steven’s code for comparison with SWEET serves to enrich our understanding of SWEET’s capabilities and performance in relation to established benchmarks and alternative methodologies. This comprehensive evaluation enables us to identify areas of excellence and opportunities for improvement, ultimately contributing to the advancement of simulation techniques in the field of light transport modeling.

**Figure 2:**
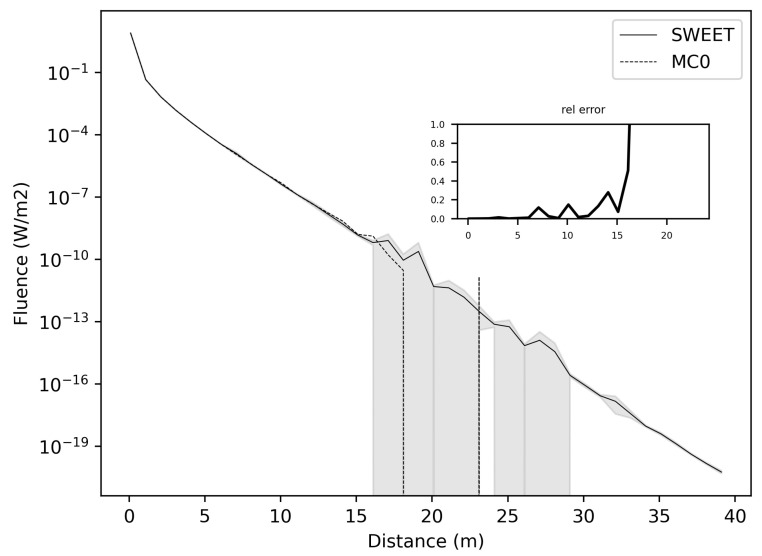
Comparing SWEET to Steven’s Monte Carlo code for a simple situation on fluence evaluation, which is computed as the integral of the radiance over the solid angles of 3D space $\int_{\Omega }L\left(r,\vec{u}\right)d\Omega$ (*L* is the radiance at position *r* and in a direction $\vec{u}$), for asymetry phase parameter: g = 0.0, optical parameters: σ = 0.5 m^−1^ and κ = 0.5 m^−1^, the 95% confidence intervals for SWEET are in grey.

This section presents the results of comparisons between SWEET and Steven’s code ([44], available https://omlc.org/classroom/ece532/class4/ssmc/index.html, accessed on 24 October 2024), followed by a comparison between SWEET and Mitsuba ([4]). In all the figures presented, “MC0” stands for the Steven’s Monte Carlo code and “MS” stands for Mitsuba. The choice of test parameters in this paper is not arbitrary. Our focus is on the primary optical parameters that play a central role in the calculation of radiometric quantities such as luminance and fluence. Here, the key parameters are the optical properties of the medium: specifically, extinction, scattering coefficient, absorption, and the anisotropy of the medium, characterized by the asymmetry parameter of the phase function. There are indeed many additional parameters that could be considered, notably those related to the scene configuration, the characteristics of the light sources used, and the specific attributes of the modeled sensors (such as camera model parameters). However, the objective of this paper is to provide an initial stage of validation that focuses on evaluating the modeling of light propagation within a participating medium, governed by solving the radiative transfer equation. To this end, we have selected simplified configurations (isotropic point sources, infinite media, a straightforward scene, etc.) and a basic point sensor. This approach enables us to isolate and assess the essential optical parameters without the added complexity of more elaborate scene and sensor configurations, thus focusing on the core validation of the light propagation model itself. In our comparisons, we considered the case of an isotropic point source placed in an infinite participating medium characterized by the Henyey-Greenstein phase function. The Figure 2 shows the comparison of SWEET and Steven’s code ([44]). In all the presented results, we considered the extinction coefficient β = 1 m^−1^. So distances are equivalent to optical thicknesses τ.

Figure 2 illustrates fluence, which is computed as the integral of the radiance over the solid angles of 3D space $\int_{\Omega }L\left(r,\vec{u}\right)d\Omega$ (*L* is the radiance at position *r* and in a direction $\vec{u}$). This figure shows minimal discrepancies between SWEET and MC0 (and the confidence intervals for SWEET in grey). However, beyond a distance of 20 m corresponding to an optical tickness of 20 since β = 1 m^−1^), the MC0 simulator exhibits a lack of precision, yielding zero values. Conversely, with SWEET, the fluence continues to attenuate even after the 20-m mark, albeit with some observed uncertainties (as evidenced by a slight widening of the confidence interval). This suggests that while both simulators perform reasonably well in capturing fluence levels within shorter distances, SWEET demonstrates greater robustness and accuracy in simulating the attenuation of fluence over longer distances. The persistence of fluence attenuation with SWEET, coupled with the widening confidence intervals, indicates a more nuanced understanding of the light transport phenomena, allowing for better characterization of uncertainties and more reliable predictions even in challenging scenarios. These findings underscore the advantage of SWEET in providing accurate and consistent simulations for a wide range of distances, making it a valuable tool for applications requiring precise modeling of light propagation in complex environments.

**Figure 3:**
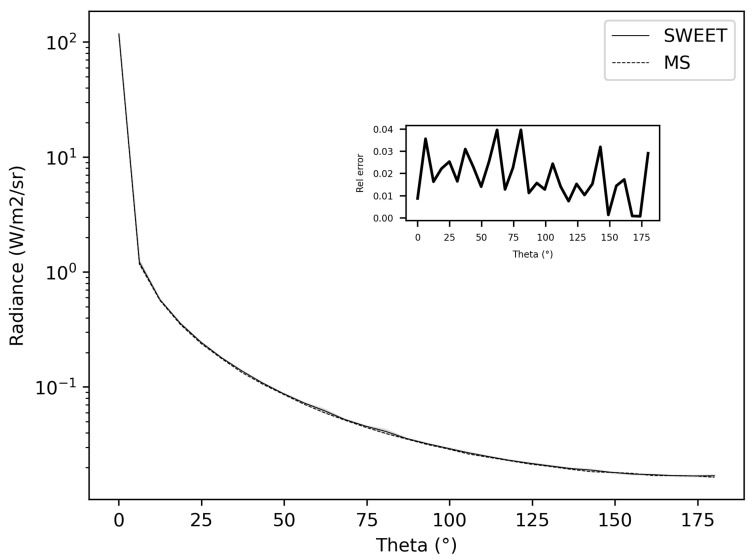
Comparing SWEET to Mitsuba for a simple situation on radiance evaluation at a distance of 1 mm, for asymetry phase parameter: g = 0.5, optical parameters: σ = 0.9 m^−1^ and κ = 0.1 m^−1^.

Figure 3 depicts differences not exceeding 4% between SWEET and MS at a distance of 1 mm from the point source. As we delve further, we will showcase comparisons for additional distances to elucidate potential limitations and variances.

The initial comparison between SWEET and MS/MC0 yields promising results, suggesting a close agreement in modeling light propagation dynamics, as evidenced by the Figure 2 and Figure 3 However, to comprehensively gauge the performance and applicability of these simulations, it’s essential to broaden the scope of analysis. This entails exploring a range of distances from the source, encompassing both near-field and far-field scenarios. By doing so, we can capture the all kinds of light propagation behaviors and assess how well the models perform across various spatial extents. Additionally, delving into different optical parameters such as absorption (κ) and scattering (σ) coefficients and the anisotropy phase parameter (g) is crucial. These parameters play a significant role in dictating the interaction of light with the medium, and understanding their impact on simulation outcomes is vital for real-world applications. Through this comprehensive analysis, we aim to not only validate the agreement between SWEET and MS/MC0 but also to elucidate the factors that influence their performance, thus enhancing their utility across a wide range of practical applications in fields such as automotive perception, biomedical imaging, environmental monitoring, and materials science.

**Figure 4:**
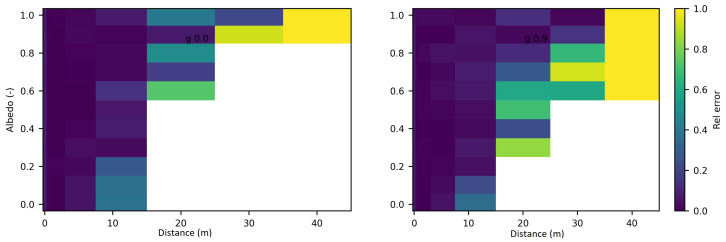
Relative discrepancies for fluence between SWEET to Steven’s Monte Carlo code for a set of distances, optical coeficients (κ & σ) and anisotropy phase parameter (g). Albedo $=\frac{\sigma }{\sigma +\kappa }$, where β=σ+κ is taken equal to 1.0; g = 0 in the **left** and g = 0.9 in the **right**.

**Figure 5:**
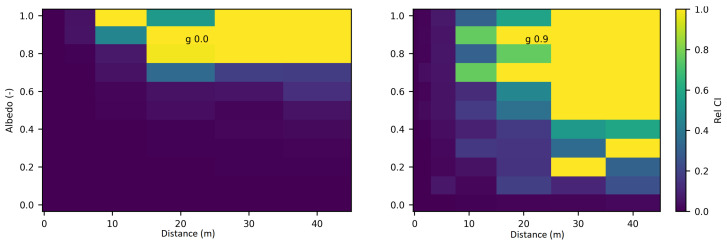
Relative 95% confidence intervals of SWEET for fluence for a set of distances, optical coeficients (κ & σ) and anisotropy phase parameter (g). Albedo $=\frac{\sigma }{\sigma +\kappa }$, g = 0 in the **left** and g = 0.9 in the **right**.

Figure 4 displays the relative discrepancies resulting from the comparison between SWEET and MC0 for two values of the phase asymmetry parameter. For a value of g = 0 (indicating an isotropic phase function), it is observed that the differences are minimal (less than 10%) for distances less than 10 m as seen in Table 4. However, beyond 10m, the discrepancies become relatively substantial, with white regions indicating instances where MC0 provided a null value. Nevertheless, as depicted in Figure 5, SWEET continues to provide results even as its confidence interval widens. This observation has already been noted through Figure 2. We can also observe through Table 4 that several values provided by MC0 are not very consistent (notably, the average value of $9.34\times 10^{-3}$ is higher than the previous row, which is illogical and implies a divergence in the results with MC0 for high distances). This may stem from the calculation method used by the MC0 code, which requires refining the step size between calculation points to increase result accuracy, at the cost of drastically increasing computation time. Unfortunately, we do not have the standard deviations of this code to verify the convergence.

Furthermore, it’s evident that as the distance increases and the albedo increases, the discrepancies between SWEET and MC0 become more pronounced, the exceed 100% as seen in Figure 4 and the red colored relative errors in Table 4. Conversely, the differences are relatively minor when the albedo is low. Another observation from comparing the right and left Figure 4 is that the discrepancies are smaller for larger distances when the albedo is high and the medium is diffusing in the forward direction (i.e., g = 0.9).

These findings underscore the complex interplay between distance, albedo, and the accuracy of light propagation modeling techniques. The widening of the confidence interval for SWEET at greater distances (as seen in Table 4) suggests a decrease in confidence in its predictions, likely due to the increasing complexity of light interactions with the medium over longer propagation paths. Additionally, the correlation between discrepancies and albedo implies that the accuracy of the models is influenced by the scattering properties of the medium. In future work, it would be interesting to study the correlation between various optical parameters to analyze a potential multivariate trend.

Understanding these relationships is crucial for refining simulation models and improving their predictive capabilities, particularly in scenarios where accurate estimation of light propagation is essential, such as in automotive applications. Further investigation into the underlying mechanisms driving these observations is warranted to enhance the robustness and applicability of light propagation simulation tools across diverse real-world scenarios.

**Figure 6:**
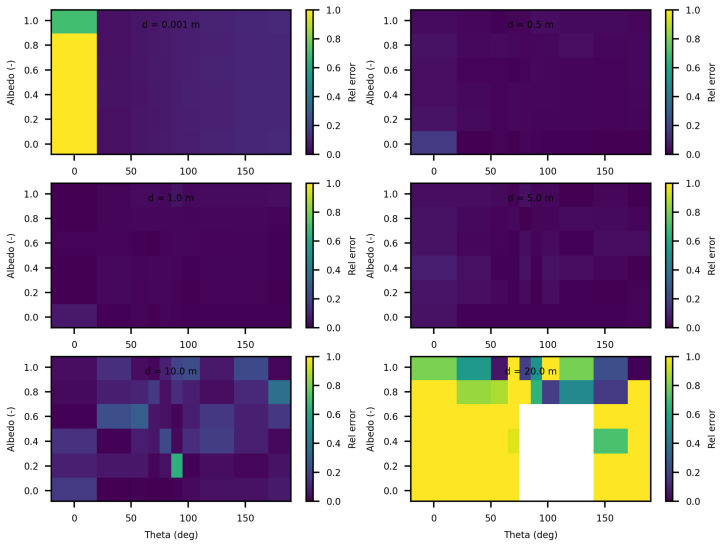
Comparing SWEET to Mitsuba Monte Carlo code for luminance for a set of distances and optical coeficients (κ & σ). Anisotropy phase parameter (g) equals to 0.0, for the case of punctual light source. Albedo $=\frac{\sigma }{\sigma +\kappa }$, where β=σ+κ is taken equal to 1.0, each figure corresponds to a distance as in this matrix: $\left[\begin{array}{cc} 0.001 & 0.5\\ 1.0 & 5.0\\ 10.0 & 20.0 \end{array}\right]$ m.

**Figure 7:**
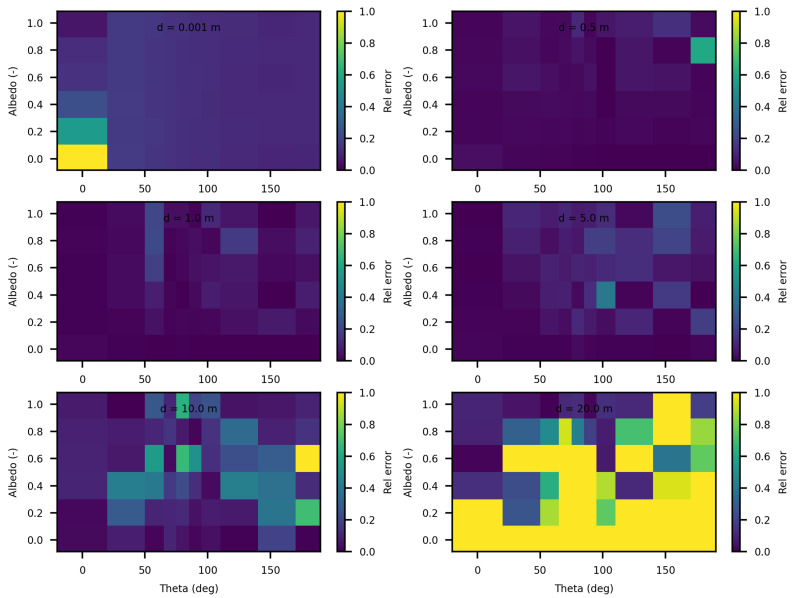
Comparing SWEET to Mitsuba Monte Carlo code for luminance for a set of distances and optical coeficients (κ & σ). Anisotropy phase parameter (g) equals to 0.9, for the case of punctual light source. Albedo $=\frac{\sigma }{\sigma +\kappa }$, where β=σ+κ is taken equal to 1.0, each figure corresponds to a distance as in this matrix: $\left[\begin{array}{cc} 0.001 & 0.5\\ 1.0 & 5.0\\ 10.0 & 20.0 \end{array}\right]$ m.

Figure 6 and Figure 7 present the relative differences between SWEET and MS for two values of the asymmetry parameter g, specifically 0 and 0.9. The data reveals that these discrepancies begin to exceed 20% when the distance from the source surpasses 10 m. This indicates a growing divergence between the two simulators’ results as one moves further away from the point source. This can be confirmed through the relative differences shown in red in Table 5. However, as it can be seen in the same table, these differences decrease as scattering coefficient increases.

Additionally, the calculations performed very close to the source, particularly at a distance of 1 mm, exhibit significant instability as shown through relative errors for small distances colored in red in Table 5. This instability arises due to the nature of the point source being modeled as a Dirac delta function. A Dirac delta function represents an idealized point source that is mathematically challenging to handle because it involves infinite intensity at a single point while having an integral over space that is finite. This characteristic leads to substantial numerical difficulties and potential inaccuracies in the simulations.

**Figure 8:**
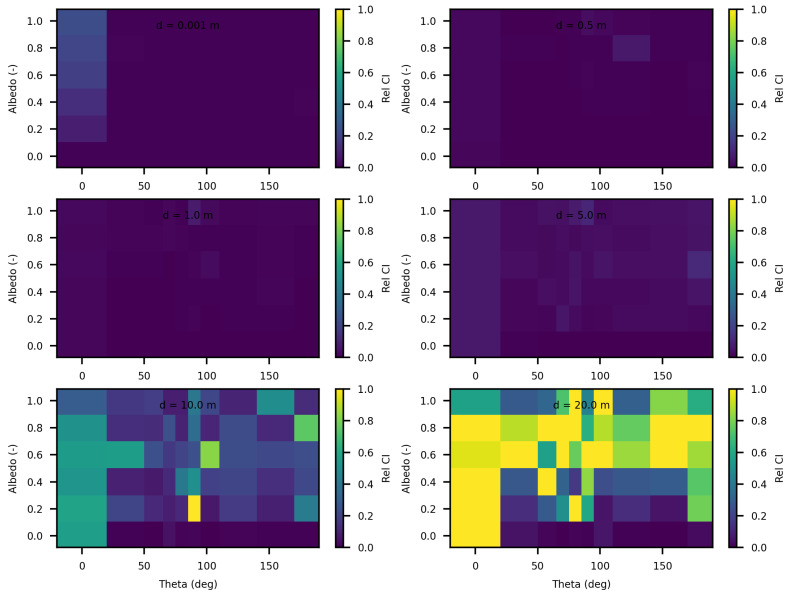
SWEET relative 95% confidence interval for luminance for a set of distances and optical coeficients (κ & σ). Anisotropy phase parameter (g) equals to 0.0, for the case of punctual light source. Albedo $=\frac{\sigma }{\sigma +\kappa }$, where β=σ+κ is taken equal to 1.0, each figure corresponds to a distance as in this matrix: $\left[\begin{array}{cc} 0.001 & 0.5\\ 1.0 & 5.0\\ 10.0 & 20.0 \end{array}\right]$ m.

**Figure 9:**
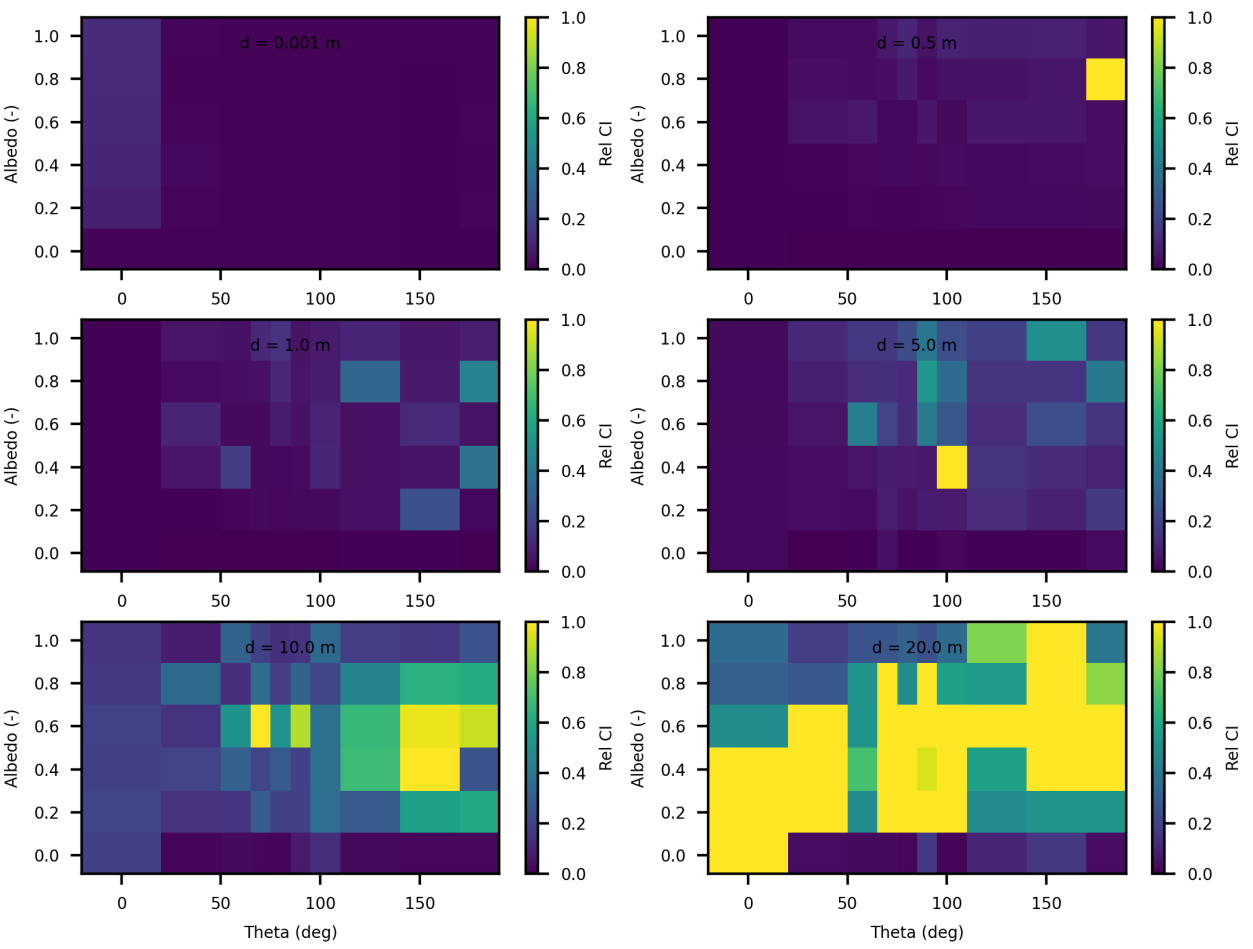
SWEET relative 95% confidence interval for luminance for a set of distances and optical coeficients (κ & σ). Anisotropy phase parameter (g) equals to 0.9, for the case of punctual light source. Albedo $=\frac{\sigma }{\sigma +\kappa }$, where β=σ+κ is taken equal to 1.0, each figure corresponds to a distance as in this matrix: $\left[\begin{array}{cc} 0.001 & 0.5\\ 1.0 & 5.0\\ 10.0 & 20.0 \end{array}\right]$ m.

The analysis of the relative confidence intervals of SWEET reveals significant uncertainties at a distance of 1 mm when the angle theta is zero as depicted in Figure 8 and Figure 9, espicially for high albedo values (high scattering medium). The relative confidence interval is about 30% for σ = 0.99 m^−1^ (computed from Table 5). Indeed, as scattering increases, there is a higher likelihood of photons being located very close to the source, which induces significant variance, as the luminance of a point source is inversely proportional to the square of the distance. Once again, it is the Dirac-like nature of this type of source that causes these difficulties. These challenges are inherent to the mathematical complexities and numerical instabilities associated with modeling such a highly localized and intense source. Beyond this immediate proximity to the source, the SWEET simulator exhibits varying levels of uncertainty, which can surpass 20% at distances greater than 10 m. This variability indicates that while SWEET is a robust tool for many applications, it struggles with precision over extended ranges when dealing with a point source.

**Figure 10:**
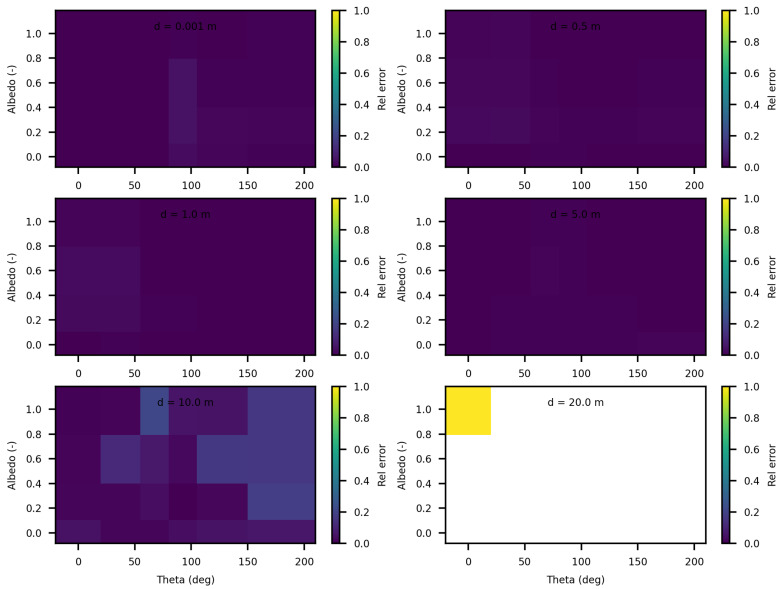
Comparing SWEET to Mitsuba Monte Carlo code for luminance for a set of distances and optical coeficients (κ & σ). Anisotropy phase parameter (g) equals to 0.0, for the case of rectangular light source. Albedo $=\frac{\sigma }{\sigma +\kappa }$, where β=σ+κ is taken equal to 1.0, each figure corresponds to a distance as in this matrix: $\left[\begin{array}{cc} 0.001 & 0.5\\ 1.0 & 5.0\\ 10.0 & 20.0 \end{array}\right]$ m.

**Figure 11:**
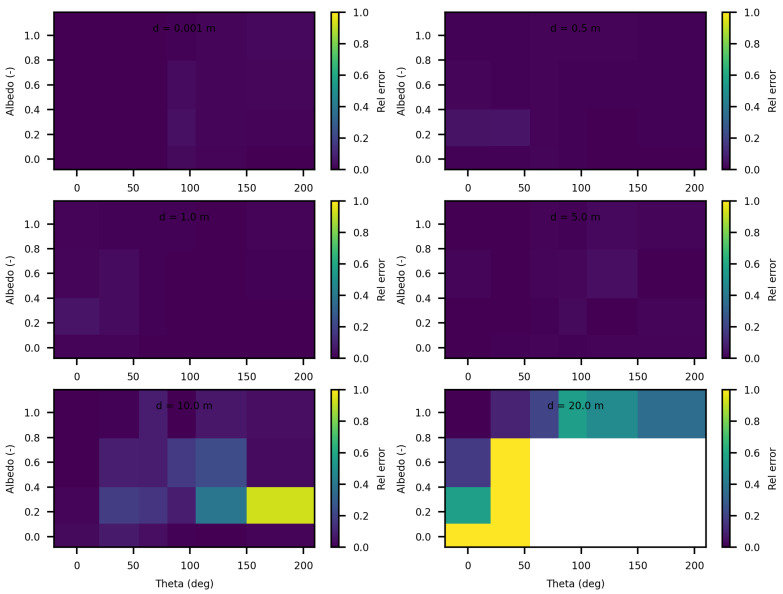
Comparing SWEET to Mitsuba Monte Carlo code for luminance for luminance for a set of distances and optical coeficients (κ & σ). Anisotropy phase parameter (g) equals to 0.9, for the case of rectangular light source. Albedo $=\frac{\sigma }{\sigma +\kappa }$, where β=σ+κ is taken equal to 1.0, each figure corresponds to a distance as in this matrix: $\left[\begin{array}{cc} 0.001 & 0.5\\ 1.0 & 5.0\\ 10.0 & 20.0 \end{array}\right]$ m.

Figure 10 and Figure 11 illustrate the luminance results for a rectangular light source measuring 2 m by 2 m, placed within a rectangular tunnel with dimensions of 2 m by 2 m by 80 m. Unlike the point source scenario discussed previously, the luminance calculations for this surface source are significantly more stable. The stability can be attributed to the more distributed nature of the light source, which alleviates the numerical difficulties associated with the Dirac delta function used to model a point source.

In this setup, the discrepancies between the luminance values calculated by SWEET and MS do not exceed 5% up to a distance of 5 m from the source. This indicates a high degree of agreement between the two methods within this range, suggesting that both simulators handle the surface source scenario effectively in close to mid-range distances. For the luminance computed in the forward direction ($L(\theta =0^{\circ })$), the differences do not exceed 5% even at a 10 m distance (Table 6).

However, beyond the 5-m mark, the differences begin to grow. At a distance of 20 m from the source, the MS method yields zero luminance values, as it can be seen in Figure 10 and Figure 11 and in Table 6. This suggests a limitation in the MS approach’s ability to accurately simulate luminance at greater distances within the tunnel environment. The tunnel’s geometry and the extended range likely contribute to the attenuation and scattering effects that challenge the MS simulator, leading to these null values. In contrast, SWEET continues to provide non-zero luminance values, indicating its robustness in handling longer-range light propagation in this specific environment. These conclusions need to be discussed further, especially regarding meaningfull optical depths in the automotive driving context.

Overall, these results underscore the advantages of using a surface source model over a point source model in terms of computational stability and accuracy in practical applications.

**Figure 12:**
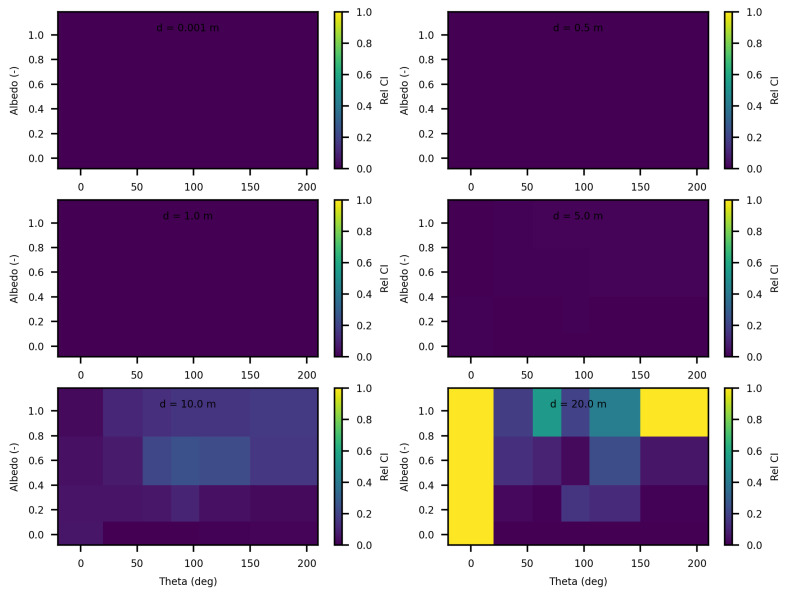
SWEET relative 95% confidence interval for a set of distances and optical coeficients (κ & σ). Anisotropy phase parameter (g) equals to 0.0, for the case of rectangular light source. Albedo $=\frac{\sigma }{\sigma +\kappa }$, where β=σ+κ is taken equal to 1.0, each figure corresponds to a distance as in this matrix: $\left[\begin{array}{cc} 0.001 & 0.5\\ 1.0 & 5.0\\ 10.0 & 20.0 \end{array}\right]$ m.

**Figure 13:**
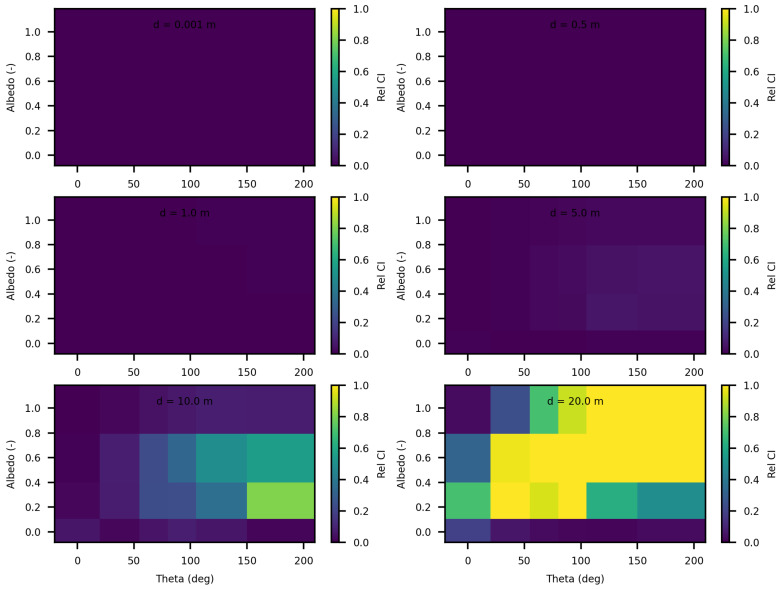
SWEET relative 95% confidence interval for luminance for a set of distances and optical coeficients (κ & σ). Anisotropy phase parameter (g) equals to 0.9, for the case of rectangular light source. Albedo $=\frac{\sigma }{\sigma +\kappa }$, where β=σ+κ is taken equal to 1.0, each figure corresponds to a distance as in this matrix: $\left[\begin{array}{cc} 0.001 & 0.5\\ 1.0 & 5.0\\ 10.0 & 20.0 \end{array}\right]$ m.

When the source is modeled as a rectangular surface (2 m by 2 m), placed in a rectangular tunnel (2 m by 2 m by 80 m), the variability and overall uncertainties in the SWEET simulations are significantly reduced compred to the Dirac-type case. Figure 12 and Figure 13 demonstrate that for this surface source scenario, the uncertainties remain low (less than 5%) up to a distance of 5 m. This improvement is likely due to the more distributed nature of the surface source, which mitigates the extreme intensity and localization issues present in the point source model. However, as the distance from the surface source increases beyond 5 m, the uncertainties begin to rise again, exceeding 10% at greater distances. This increase is expected as the challenges of accurately modeling light propagation and scattering in a confined tunnel environment become more pronounced with distance. Ongoing work on bi-directional path tracing would help with these kinds of cases with special lighting.

**Figure 14:**
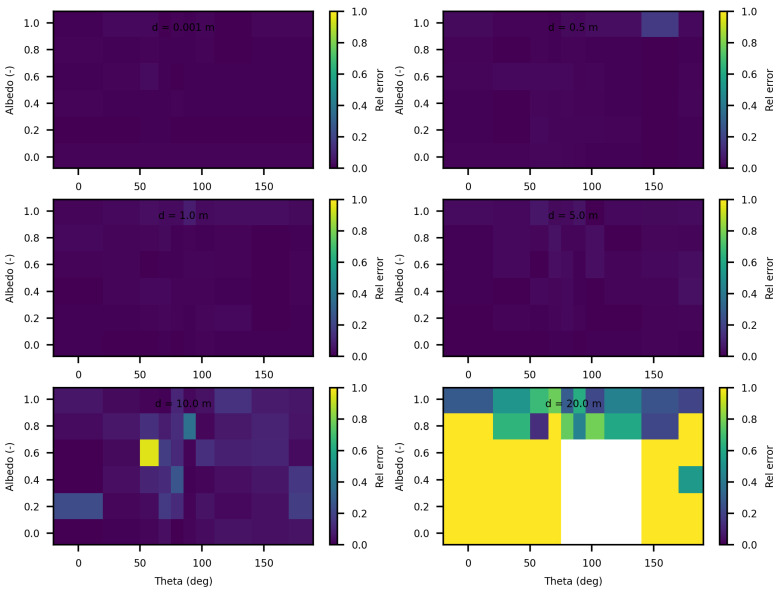
Comparing SWEET to Mitsuba Monte Carlo code for luminance for a set of distances and optical coeficients (κ & σ). Anisotropy phase parameter (g) equals to 0.0, for the case of 2 point lights. Albedo $=\frac{\sigma }{\sigma +\kappa }$, where β=σ+κ is taken equal to 1.0, each figure corresponds to a distance as in this matrix: $\left[\begin{array}{cc} 0.001 & 0.5\\ 1.0 & 5.0\\ 10.0 & 20.0 \end{array}\right]$ m.

**Figure 15:**
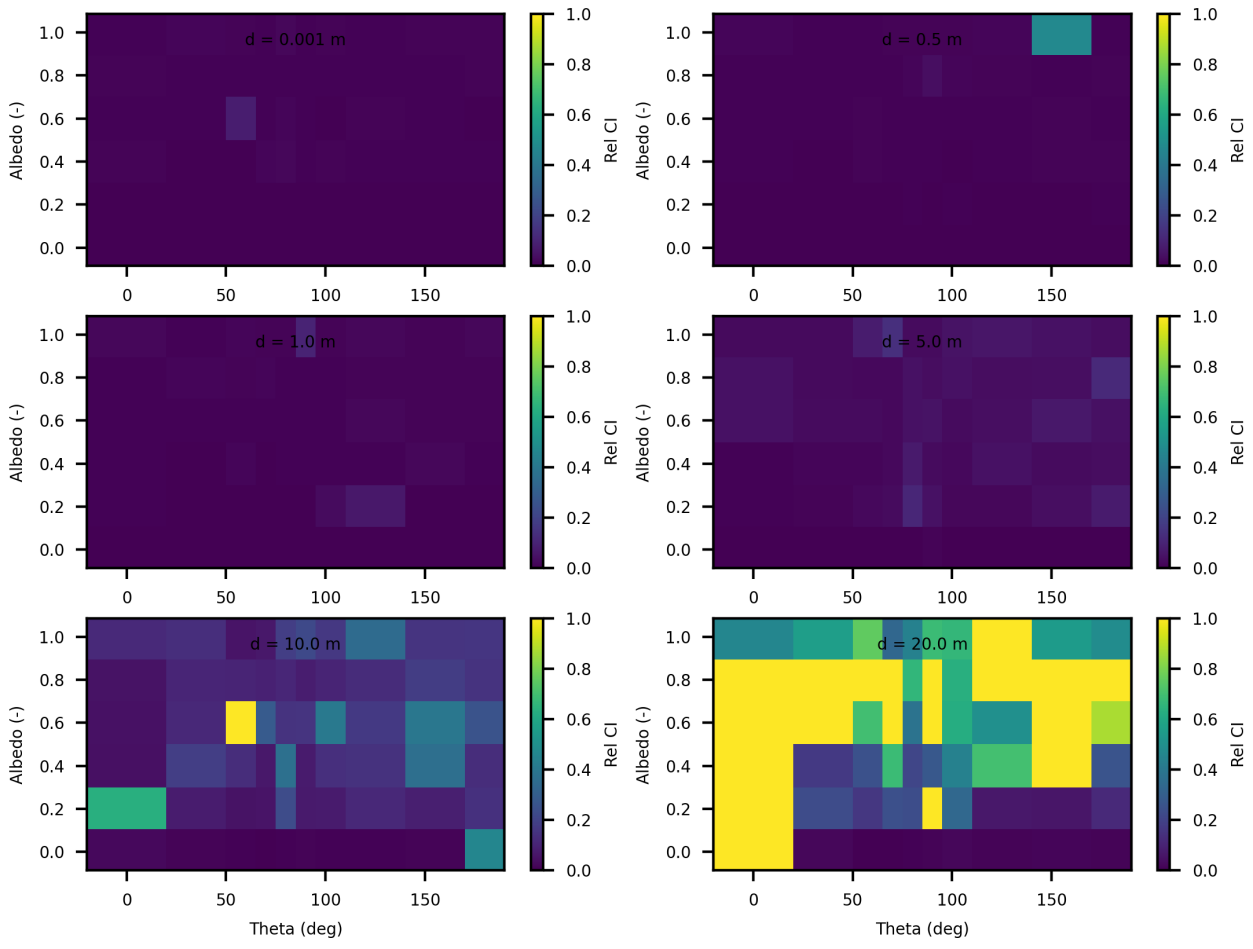
SWEET relative 95% confidence interval for luminance for a set of distances and optical coeficients (κ & σ). Anisotropy phase parameter (g) equals to 0.0, for the case of 2 point lights. Albedo $=\frac{\sigma }{\sigma +\kappa }$, where β=σ+κ is taken equal to 1.0, each figure corresponds to a distance as in this matrix: $\left[\begin{array}{cc} 0.001 & 0.5\\ 1.0 & 5.0\\ 10.0 & 20.0 \end{array}\right]$ m.

We have also added a comparison between the SWEET and MS simulators for the case of two point sources placed in an infinite medium. Figure 14 shows the relative discrepancies between the two simulators. It can be observed that these discrepancies are minor, not exceeding 5% overall up to a distance of 5 m. The higher discrepancies are attributed to the uncertainties of SWEET, as illustrated in Figure 15. Indeed, Table 7 also shows relatively wide confidence intervals at the 20 m distance.

### 4.2. Invariance Property (IP) Estimates

**Figure 16:**
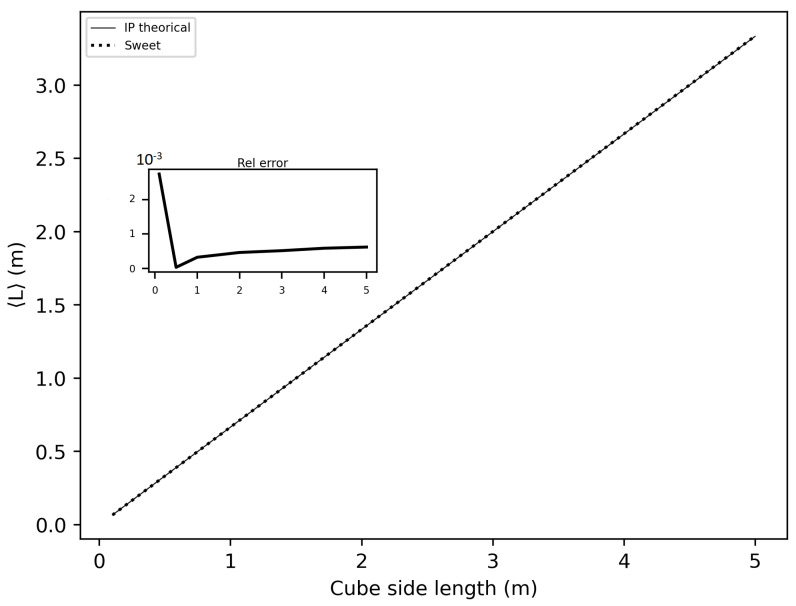
Mean path length estimated with SWEET and theorically (using IP) for cubes with side lengths ranging from 10 cm to 5 m, for asymetry phase parameter: g = 0.0, optical parameters: σ = 1.0 m^−1^ and κ = 0.0 m^−1^.

**Figure 17:**
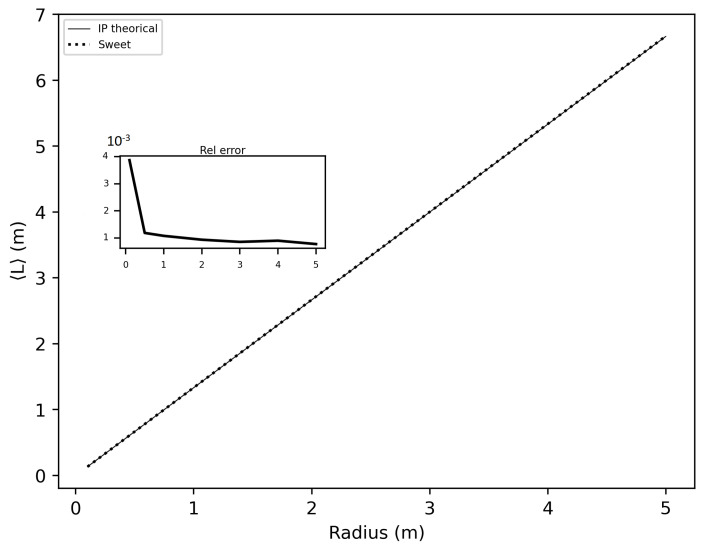
Mean path length estimated with SWEET and theorically (using IP) for spheres with radii ranging from 10 cm to 5 m, for asymetry phase parameter: g = 0.0, optical parameters: σ = 1.0 m^−1^ and κ = 0.0 m^−1^.

In this section of the article, we considered several spheres with radii ranging from 10 cm to 5 m, and cubes with sides ranging from 10 cm to 5 m. We estimated the invariance property using SWEET. Initially, we considered the following optical properties in these enclosed media: σ = 1.0 m^−1^, g=1.0 and κ = 0.0 m^−1^ to adhere to the conditions required for the application of the invariance property (IP). Figure 16 and Figure 17 show the obtained results. A relative error was calculated with respect to the theoretical IP equation, which states that for a homogeneous medium $\langle L\rangle =4\frac{V}{S}$, where 〈L〉 is the mean path length, *V* is the volume, and *S* is the surface area. For a cube, this gives $\langle L\rangle =\frac{2}{3}C$, where *C* is the side length of the cube, and for a sphere, $\langle L\rangle =\frac{4}{3}r$, where *r* is the radius of the sphere.

As shown in these figures, the relative error is negligible. The error is slightly higher in the case of spheres because SWEET models a sphere as a set of triangular facets. To further reduce this error, one could increase the number of rings and segments of the sphere, resulting in more triangles.

**Figure 18:**
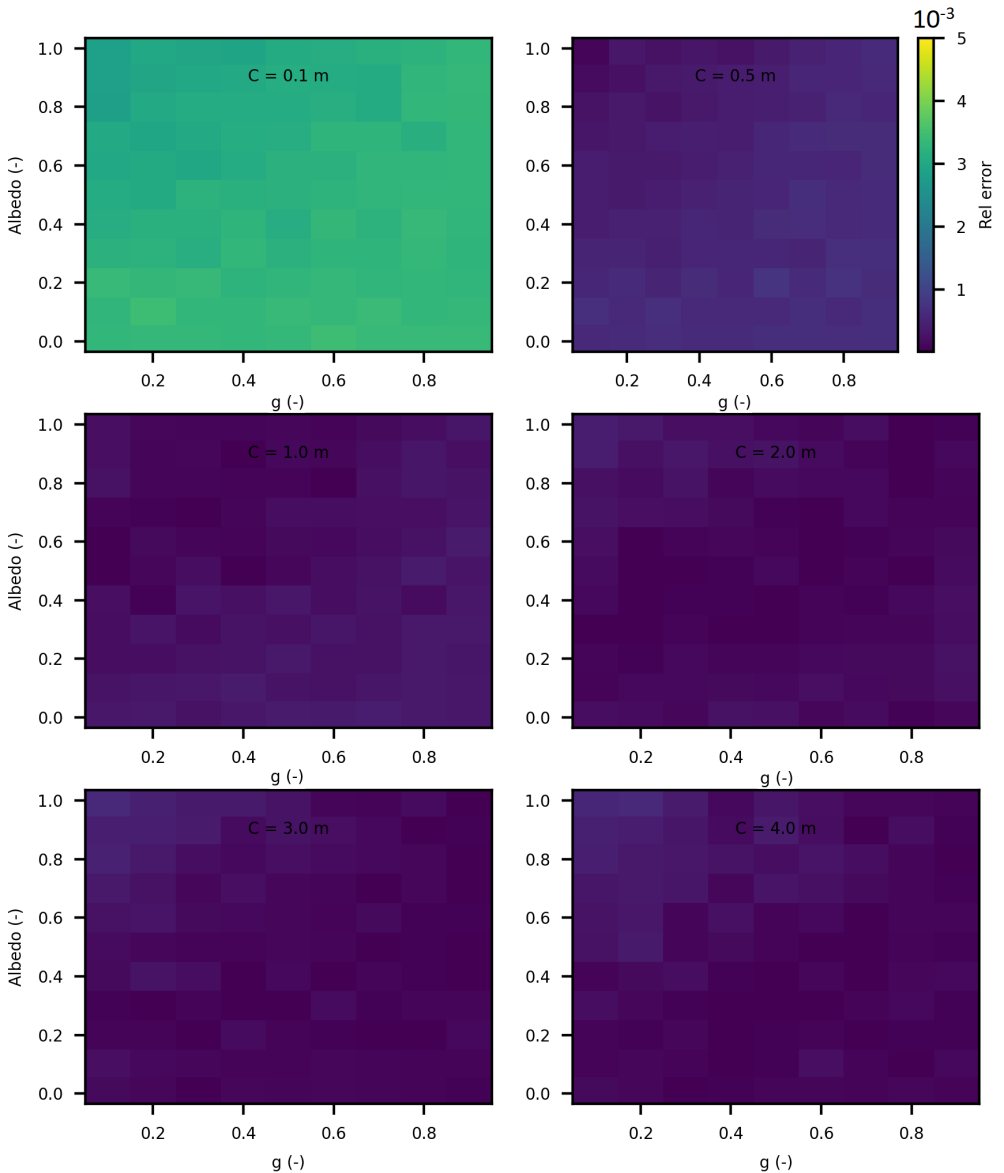
Relative errors of invariance property (IP) estimated with SWEET and theorically for cubes with side lengths ranging from 10 cm to 4 m, for varying asymetry phase parameter g and optical parameters (σ and κ), each figure corresponds to a side length as in this matrix: $\left[\begin{array}{cc} 0.1 & 0.5\\ 1.0 & 2.0\\ 3.0 & 4.0 \end{array}\right]$ m.

**Figure 19:**
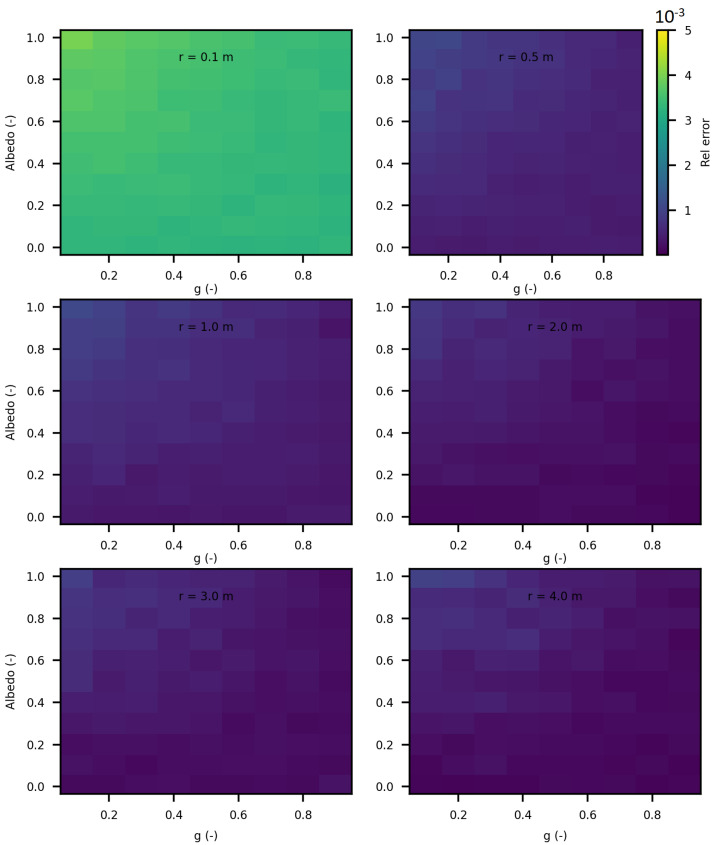
Relative errors of invariance property (IP) estimated with SWEET and theorically for spheres with radii ranging from 10 cm to 4 m, for varying asymetry phase parameter g and optical parameters (σ and κ), each figure corresponds to a radius as in this matrix: $\left[\begin{array}{cc} 0.1 & 0.5\\ 1.0 & 2.0\\ 3.0 & 4.0 \end{array}\right]$ m.

We further extended the analysis of the invariance property (IP) by varying the optical parameters (σ, κ and g) for different sizes of cubes and spheres. Figure 18 and Figure 19 display the relative errors of SWEET compared to the theoretical equation presented earlier. The results clearly demonstrate that the relative error of the IP remains low across all these estimations (not exceeding 4‰), regardless of the variations in optical parameters and geometric dimensions. However, slightly higher relative errors are observed for smaller dimensions (r = 0.1 m and C = 0.1 m). This can be attributed to the fact that SWEET uses triangular facets, which can introduce rounding errors in the approximations. As the size of the cubes and spheres decreases, the number of triangular facets increases relative to the surface area, leading to more pronounced discretization effects. These effects are more significant in smaller geometries, resulting in higher relative errors. Despite this, the errors remain within an acceptable range, indicating that SWEET is still reliable for small-scale simulations, though caution should be exercised and adjustments may be needed for higher precision. The number of Monte Carlo iterations *N* can also impact these results; increasing *N* reduces the standard deviation of the Monte Carlo results then reduces the relative error. With the computational resources at our disposal, using a Linux machine equipped with an NVIDIA RTX A4500 graphics card, we were able to increase *N* up to $1.2\times 10^{8}$. This substantial number of iterations helps to further minimize the relative error, enhancing the accuracy and reliability of the SWEET simulations even for smaller dimensions and varying optical parameters. The high iteration count ensures that statistical noise is reduced, leading to more precise estimations of the invariance property.

This analysis confirms the robustness of the SWEET tool in estimating the invariance property, even in geometrically complex shapes. By considering the conditions to adhere to the IP requirements, we can validate the accuracy and reliability of Monte Carlo simulations across a range of geometric configurations.

### 4.3. Computational Cost Analysis

In this section, we conducted a comparative analysis of the execution time between SWEET and MS. We did not include execution time comparisons with Steven’s code (MC0) because it is designed to compute fluence for multiple distances simultaneously. This approach differs from the calculation scheme used by SWEET and MS, which are specifically configured to calculate luminance at a single point and in a given direction. Consequently, a direct comparison would not be meaningful due to the inherent differences in computational methods.

The current analysis specifically applies to the case of an isotropic point source placed within an infinite medium. We examined how execution time varies according to different optical parameters. These calculations were performed on the same machine equipped with an NVIDIA GeForce GTX 950M graphics card, and the photon count was kept identical between SWEET and MS for a fair comparison. It is important to note that the focus here is solely on execution time without consideration for convergence. In previous sections, we increased the photon counts to the limits to ensure optimal convergence of the results being compared (i.e., luminance and fluence).

**Figure 20:**
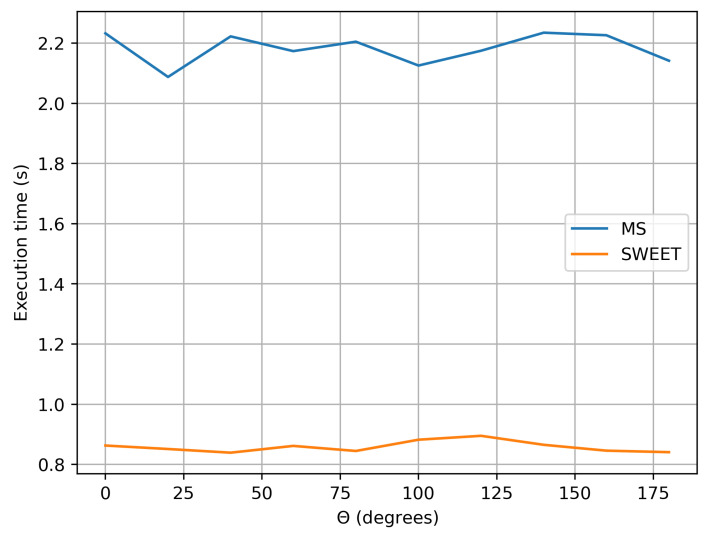
Execution time for SWEET and MS done with $10^{6}$ photons in luminance computing, for asymetry phase parameter: g = 0.9, optical parameters: σ = 0.99 m^−1^ and κ = 0.01 m^−1^.

Figure 20 illustrates the execution time of SWEET and MS for luminance calculations as a function of the theta angle in the luminance direction, with $N=10^{6}$ photons and identical optical parameters. As shown, SWEET achieves execution speeds more than twice as fast as MS in this specific case.

**Figure 21:**
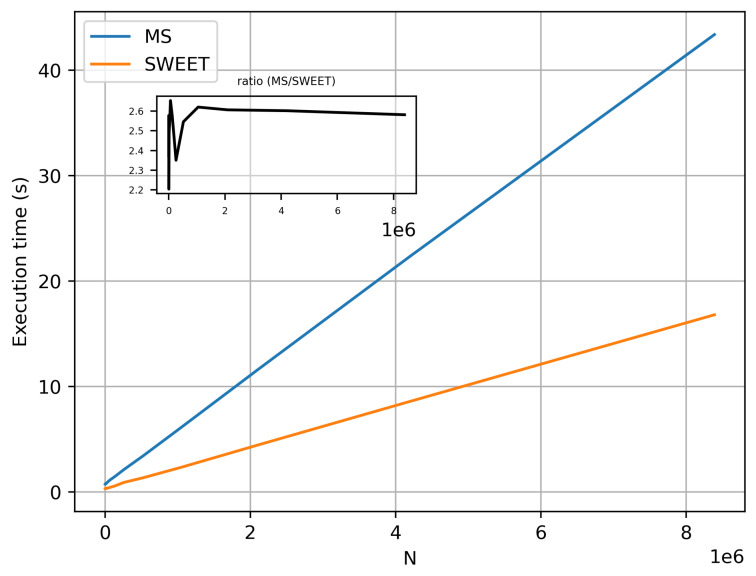
Execution time variation with the photon count for SWEET and MS for a single luminance computing (L(θ=0°)), for asymetry phase parameter: g = 0.9, optical parameters: σ = 0.99 m^−1^ and κ = 0.01 m^−1^.

Figure 21 illustrates the variations in execution time for computing the luminance L(θ=0°) for the same optical parameters as a function of the photon count, N. We observe that SWEET consistently performs at more than twice the speed of MS. We also observe that for low values of N, the ratio fluctuates significantly. This is explained by the fact that these low values of N do not allow for convergence of the luminance, leading to instability in the execution time ratio between SWEET and MS.

**Figure 22:**
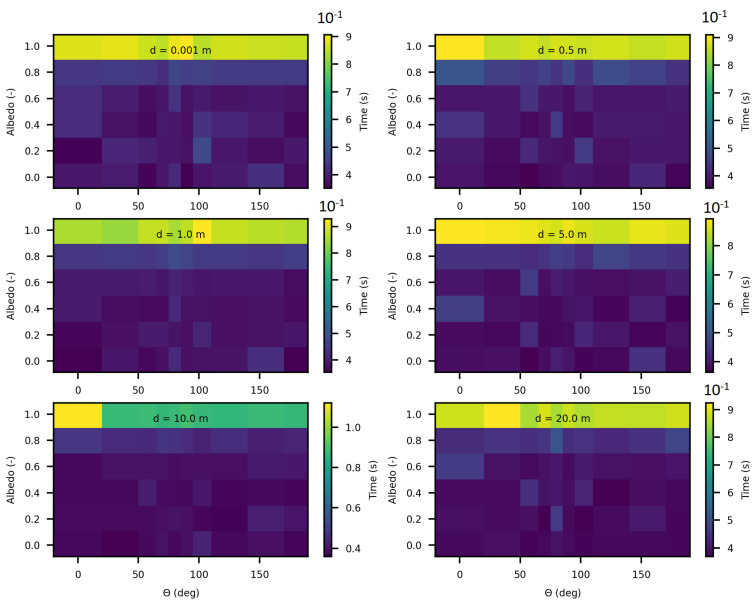
Execution time variation for SWEET for luminance computing, done with $10^{6}$ photons for varying optical parameters, for asymetry phase parameter: g = 0.9, each figure corresponds to a distance as in this matrix: $\left[\begin{array}{cc} 0.001 & 0.5\\ 1.0 & 5.0\\ 10.0 & 20.0 \end{array}\right]$ m.

**Figure 23:**
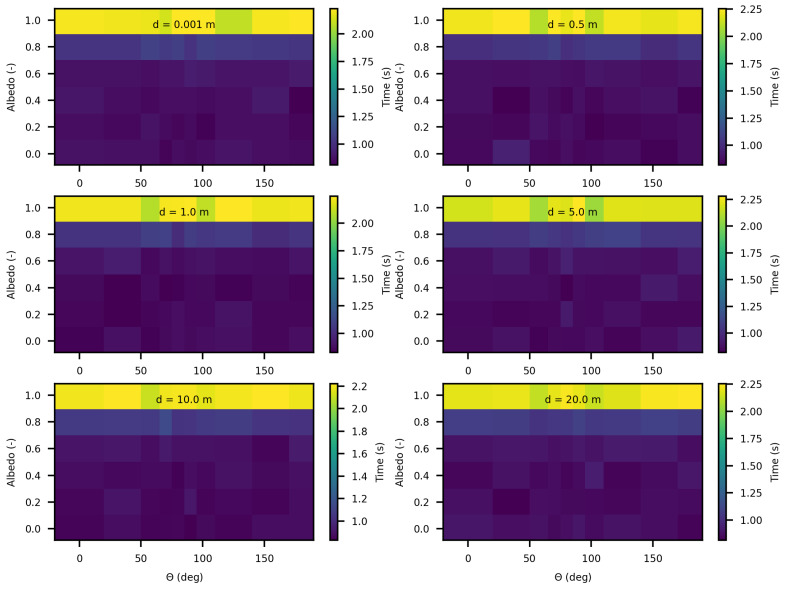
Execution time variation for MS for luminance computing, done with $10^{6}$ photons for varying optical parameters, for asymetry phase parameter: g = 0.9, each figure corresponds to a distance as in this matrix: $\left[\begin{array}{cc} 0.001 & 0.5\\ 1.0 & 5.0\\ 10.0 & 20.0 \end{array}\right]$ m.

In the following analysis, we computed execution times while varying optical parameters and the distances between the point source and the target point for luminance calculation. Figure 22 and Figure 23 present these results for SWEET and MS, respectively, with an extinction coefficient β set to 1 m^−1^ and asymetry parameter set to 0.9 across all calculations.

The results in these Figure 22 and Figure 23 reveal, first, that as expected, execution time increases as the medium becomes highly scattering (for values of albedo greater than 0.9). This is explained by the increase in the number of scattering events in highly diffusive media, which requires additional computation per photon path. In these highly scattering media, the execution time reaches approximately 0.9 s with SWEET and 2.25 s with MS.

An intriguing observation in all results shown in Figure 20, Figure 22 and Figure 23 is that execution time remains independent of the angle θ of the luminance, despite the medium being infinite and forward-scattering with an asymmetry parameter of g=0.9. This outcome is likely due to the use of an analytical delta point source, which is never physically intersected.

**Figure 24:**
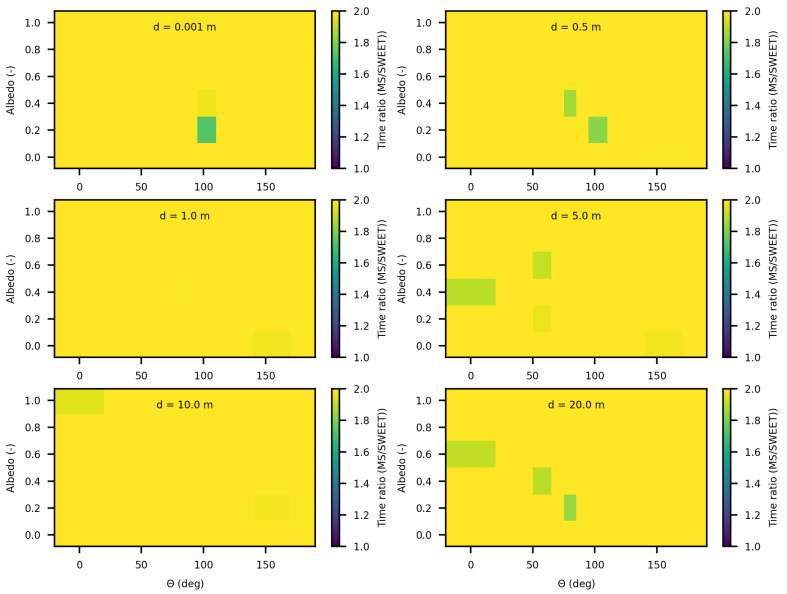
Execution time ratio variation of MS to SWEET for luminance computing, done with $10^{6}$ photons for varying optical parameters, for asymetry phase parameter: g = 0.9, each figure corresponds to a distance as in this matrix: $\left[\begin{array}{cc} 0.001 & 0.5\\ 1.0 & 5.0\\ 10.0 & 20.0 \end{array}\right]$ m.

We also observe that SWEET consistently executes in less than half the time required by MS. This finding is further supported by Figure 24, which displays the execution time ratio of MS to SWEET, confirming SWEET’s advantage in efficiency.

## 5. Discussion

The SWEET simulator has several distinct advantages that make it a valuable tool for radiative transfer and physically based imaging simulations. One of SWEET’s main strengths is its modular design, which allows for flexibility in integrating custom algorithms and features that may not be readily available in other simulators. This flexibility is especially beneficial for simulating complex environmental factors and material interactions, which are essential for autonomous vehicle applications. Additionally, SWEET performs well in terms of accuracy, especially for low optical thicknesses, even though modeling point sources remains challenging due to their Dirac-like nature as seen through Figure 6 and Figure 7. The use of surface-based light sources in SWEET also leads to faster convergence and more stable results (illustrated in Figure 10 and Figure 11), making it a reliable choice for a range of imaging tasks.

Moreover, the BSDF models implemented in SWEET enable realistic light interactions with various surfaces, which is critical for applications such as camera-based perception in automated vehicles. With microfacet models, SWEET can accurately simulate surface roughness, which enhances the realism of the rendered images.

However, SWEET also presents certain limitations. The current implementation currently struggles with modeling point sources, which exhibit Dirac-like properties that can be challenging to simulate effectively. While multiple importance sampling (MIS) has accelerated convergence in volumetric path tracing, Monte Carlo noise remains an issue that impacts the clarity of the results. To address this, bidirectional path tracing could be added to explore more complex light paths, improving SWEET’s performance in scenes with intricate lighting. Ongoing work will provide a comprehensive study on the convergence speed between different versions of SWEET, particularly comparing versions with and without importance sampling.

The analysis conducted in this article, whether comparing SWEET to MC0 or to MS, is based on the selection of key optical parameters (σ, g, optical thickness). Our approach allowed us to combine these parameters to analyze discrepancies between the results. However, there may be correlations among these parameters. Therefore, in future work, it would be valuable to study the correlation between various optical parameters to investigate a potential multivariate trend leading to better understanding the outcomes.

Another area where SWEET could advance is in the integration of time-dependent calculations. This feature would expand the simulator’s applicability to time-sensitive technologies like Lidar, which requires precise temporal information. The study of polarized light also remains an underexplored area in SWEET, and its incorporation would create new research possibilities, particularly in fields where polarization plays a role, such as meteorological estimation and material identification. These potential enhancements reflect a commitment to making SWEET not only a versatile tool for radiative transfer and imaging simulations but also one that continuously evolves to address the emerging needs of autonomous vehicle sensing and imaging applications.

## 6. Conclusions and Perspectives

In conclusion, this article presents recent advancements in the SWEET simulation environment, initially designed for radiative transfer and now adapted for physically based imaging with a focus on automated vehicles. In previous work, comparative analysis between SWEET and other simulators, such as Mitsuba, allowed for a detailed evaluation of SWEET’s image accuracy based on low-level metrics like contrast and pixel intensity.

In this paper, we proposed additional verification methods based on radiometric quantities such as luminance and fluence. We first compared the relative errors of SWEET with those of Steven’s Monte Carlo code and then compared SWEET’s results with those of MS. The comparison with MC0 results highlighted the robustness of SWEET, as it is capable of producing results even for high optical depths. However, these results are still limited by the inherent noise characteristic of Monte Carlo simulations, which can affect the accuracy of the computed radiometric quantities, as shown in Figure 5. On the other hand, the comparison with MS results demonstrated a good overall consistency between the two simulators. The largest discrepancies were observed when simulating a point source, which is inherently challenging to model due to its Dirac-like nature. This difficulty stems from the fact that point sources represent an idealized, singularity-like light source, which is particularly problematic in radiative transfer simulations. As a result, the simulations of point sources in SWEET, as well as in other simulators like MS, tend to exhibit higher errors compared to surface-based sources, which are easier to handle due to their continuous distribution of radiance. Despite these challenges, the overall agreement between SWEET and MS suggests that SWEET’s implementation is generally reliable for a wide range of simulations. On the other hand, SWEET achieves a good convergence and more stable results when using surface-based light sources as seen through Figure 12 and Figure 13. Furthermore, the use of the invariance property has yielded conclusive results for SWEET, proving that there are no implementation issues or improper handling of boundary conditions (Figure 18 and Figure 19). The comparative analysis of computational costs highlighted SWEET’s speed advantage, showing that it is over twice as fast as MS for the presented case study (Figure 24). This efficiency positions SWEET as a promising tool for simulations requiring high-speed processing without compromising accuracy.

Looking ahead, there are several exciting directions for future development of SWEET. The integration of BSDF models within SWEET has significantly enhanced its ability to simulate realistic light interactions, making it particularly well-suited for automotive imaging applications, where accurate rendering is critical for perception systems. While recent improvements, such as Multiple Importance Sampling (MIS), have notably accelerated convergence in volumetric path tracing, challenges such as Monte Carlo noise persist. One promising future enhancement is the integration of bidirectional path tracing, which could help reduce this noise and improve convergence across a wider range of geometric configurations. Furthermore, future development will focus on incorporating time-dependent calculations, which would be invaluable for applications like Lidar, as well as the modeling of polarized light, which is particularly relevant for meteorological studies. These advancements will not only broaden SWEET’s applicability in various domains but also strengthen its role as a powerful tool for both radiative transfer and imaging simulations, particularly in the context of automated driving perception systems.

## Figures and Tables

**Table 1:** Optical thickness obtained for different limit cases in the automotive context, with the fog analysis.

	Light fog on Highway	Dense Fog on Road	Very Heavy Fog on Roa	PAVIN Fog and Rain Facility Maximum Capability
Vehicle speed (km/h)	130	50	50	
Minimal detection distance (m)	108	42	42	50
MOR (m)	400	50	10	8
Optical thickness	0.8	2.5	12.5	18.8

**Table 2:** Optical thickness obtained for different limit cases in the automotive context, with sensors analysis.

		Camera vs. Sun	Camera vs. Headlamps	Cerema’s Sensors Capabilities
**Sensor**	**Minimal radiance**	6.51 × 10^−3^ cd/m^2^	6.51 × 10^−3^ cd/m^2^	3.25 × 10^−7^ W/sr/cm^2^
	**Optical aperture**	70Â°	70Â°	4Â°
	**Minimal irradiance**	5.00 × 10^−2^ Lm/m^2^	5.00 × 10^−2^ Lm/m^2^	1.43 × 10^−7^ W/cm^2^
**Source**	**Maximal Power**		2.15 × 10^5^ cd	4.00 × 10^3^ Lm
	**Surface**		6.40 × 10^−3^ m^2^	1.30 × 10^3^ cm^2^
	**Maximal irradiance**	2.00 × 10^5^ lux		4.52 × 10^−3^ W/cm^2^
	**Maximal radiance**	6.37 × 10^4^ cd/m^2^	3.36 × 10^7^ cd/m^2^	
**Ratio sensor / source**		**1.02 × 10^−7^**	**1.94 × 10^−10^**	**3.15 × 10^−5^**
**Resultant optical thickness**		**16.1**	**22.4**	**10.4**

**Table 3:** Summary of BSDF models in SWEET.

Model	Common Name	Microfacet Model (If Applicable)
Diffuse	Lambertian	N/A
Smooth Dielectric	Glass, Water	N/A
Thin Dielectric	Soap Bubble, Thin Film	N/A
Rough Dielectric	Frosted Glass, Rough Plastic	Beckmann, GGX
Smooth Conductor	Polished Metal	N/A
Rough Conductor	Brushed Metal	Beckmann, GGX
Plastic	Glossy Plastic	N/A

**Table 4:** Comparing fluence with SWEET and Steven’s Monte Carlo code for σ={0.01,0.5,0.99} m^−1^, g = {0,0.9} and a set of distances, the mean values of SWEET and MC0 and the 95% confidence intervals are shown and the relative differences, fluence values are in (W/m^2^), red-colored values are for relatively high discrepancies, - are for points where MC0 gives a null value.

Distance (m)	g = 0.0					g = 0.9				
	Mean SWEET	Mean MC0	Inf SWEET	Sup SWEET	Rel Error	Mean SWEET	Mean MC0	Inf SWEET	Sup SWEET	Rel Error
σ = 0.01 m^−1^										
0.001	$7.95\times 10^{+04}$	$7.39\times 10^{+04}$	$7.95\times 10^{+04}$	$7.95\times 10^{+04}$	$7.61\times 10^{-02}$	$7.95\times 10^{+04}$	$7.39\times 10^{+04}$	$7.94\times 10^{+04}$	$7.96\times 10^{+04}$	$7.67\times 10^{-02}$
0.5	$1.95\times 10^{-01}$	$1.94\times 10^{-01}$	$1.95\times 10^{-01}$	$1.95\times 10^{-01}$	$5.08\times 10^{-03}$	$1.94\times 10^{-01}$	$1.93\times 10^{-01}$	$1.94\times 10^{-01}$	$1.94\times 10^{-01}$	$5.33\times 10^{-03}$
1.0	$2.96\times 10^{-02}$	$2.96\times 10^{-02}$	$2.96\times 10^{-02}$	$2.96\times 10^{-02}$	$4.42\times 10^{-04}$	$2.95\times 10^{-02}$	$2.96\times 10^{-02}$	$2.95\times 10^{-02}$	$2.96\times 10^{-02}$	$7.83\times 10^{-04}$
5.0	$2.20\times 10^{-05}$	$2.32\times 10^{-05}$	$2.20\times 10^{-05}$	$2.20\times 10^{-05}$	$5.07\times 10^{-02}$	$2.25\times 10^{-05}$	$2.36\times 10^{-05}$	$2.24\times 10^{-05}$	$2.25\times 10^{-05}$	$4.99\times 10^{-02}$
10.0	$3.72\times 10^{-08}$	$5.99\times 10^{-08}$	$3.72\times 10^{-08}$	$3.73\times 10^{-08}$	$3.78\times 10^{-01}$	$3.92\times 10^{-08}$	$6.05\times 10^{-08}$	$3.90\times 10^{-08}$	$3.94\times 10^{-08}$	$3.53\times 10^{-01}$
20.0	$4.21\times 10^{-13}$	$0.00\times 10^{+00}$	$4.20\times 10^{-13}$	$4.23\times 10^{-13}$	-	$8.18\times 10^{-13}$	$0.00\times 10^{+00}$	$3.15\times 10^{-13}$	$1.32\times 10^{-12}$	-
30.0	$8.51\times 10^{-18}$	$0.00\times 10^{+00}$	$8.45\times 10^{-18}$	$8.57\times 10^{-18}$	-	$9.89\times 10^{-18}$	$0.00\times 10^{+00}$	$8.80\times 10^{-18}$	$1.10\times 10^{-17}$	-
40.0	$2.16\times 10^{-22}$	$0.00\times 10^{+00}$	$2.16\times 10^{-22}$	$2.16\times 10^{-22}$	-	$2.86\times 10^{-22}$	$0.00\times 10^{+00}$	$2.06\times 10^{-22}$	$3.66\times 10^{-22}$	-
σ = 0.5 m^−1^										
0.001	$7.96\times 10^{+04}$	$7.36\times 10^{+04}$	$7.96\times 10^{+04}$	$7.96\times 10^{+04}$	$8.20\times 10^{-02}$	$7.95\times 10^{+04}$	$7.35\times 10^{+04}$	$7.94\times 10^{+04}$	$7.96\times 10^{+04}$	$8.15\times 10^{-02}$
0.5	$2.95\times 10^{-01}$	$2.95\times 10^{-01}$	$2.94\times 10^{-01}$	$2.95\times 10^{-01}$	$1.11\times 10^{-03}$	$2.51\times 10^{-01}$	$2.51\times 10^{-01}$	$2.50\times 10^{-01}$	$2.52\times 10^{-01}$	$3.19\times 10^{-04}$
1.0	$5.86\times 10^{-02}$	$5.87\times 10^{-02}$	$5.85\times 10^{-02}$	$5.87\times 10^{-02}$	$8.83\times 10^{-04}$	$4.88\times 10^{-02}$	$4.92\times 10^{-02}$	$4.85\times 10^{-02}$	$4.91\times 10^{-02}$	$7.83\times 10^{-03}$
5.0	$1.29\times 10^{-04}$	$1.29\times 10^{-04}$	$1.27\times 10^{-04}$	$1.30\times 10^{-04}$	$1.28\times 10^{-04}$	$2.54\times 10^{-04}$	$2.50\times 10^{-04}$	$2.45\times 10^{-04}$	$2.63\times 10^{-04}$	$1.40\times 10^{-02}$
10.0	$4.30\times 10^{-07}$	$4.58\times 10^{-07}$	$4.04\times 10^{-07}$	$4.55\times 10^{-07}$	$6.21\times 10^{-02}$	$4.32\times 10^{-06}$	$4.18\times 10^{-06}$	$3.63\times 10^{-06}$	$5.01\times 10^{-06}$	$3.40\times 10^{-02}$
20.0	$5.62\times 10^{-12}$	$0.00\times 10^{+00}$	$4.65\times 10^{-12}$	$6.58\times 10^{-12}$	-	$1.26\times 10^{-09}$	$4.08\times 10^{-09}$	$7.60\times 10^{-10}$	$1.76\times 10^{-09}$	$6.92\times 10^{-01}$
30.0	$9.82\times 10^{-17}$	$0.00\times 10^{+00}$	$6.65\times 10^{-17}$	$1.30\times 10^{-16}$	-	$1.00\times 10^{-13}$	$0.00\times 10^{+00}$	$3.03\times 10^{-15}$	$2.85\times 10^{-14}$	-
40.0	$2.53\times 10^{-21}$	$0.00\times 10^{+00}$	$2.53\times 10^{-21}$	$2.53\times 10^{-21}$	-	$4.10\times 10^{-17}$	$0.00\times 10^{+00}$	$2.90\times 10^{-17}$	$4.50\times 10^{-18}$	-
σ = 0.99 m^−1^										
0.001	$7.97\times 10^{+04}$	$7.47\times 10^{+04}$	$7.97\times 10^{+04}$	$7.97\times 10^{+04}$	$6.73\times 10^{-02}$	$7.96\times 10^{+04}$	$7.60\times 10^{+04}$	$7.95\times 10^{+04}$	$7.97\times 10^{+04}$	$4.65\times 10^{-02}$
0.5	$5.94\times 10^{-01}$	$5.93\times 10^{-01}$	$5.92\times 10^{-01}$	$5.95\times 10^{-01}$	$7.22\times 10^{-04}$	$3.28\times 10^{-01}$	$3.36\times 10^{-01}$	$3.25\times 10^{-01}$	$3.30\times 10^{-01}$	$2.45\times 10^{-02}$
1.0	$2.20\times 10^{-01}$	$2.21\times 10^{-01}$	$2.19\times 10^{-01}$	$2.20\times 10^{-01}$	$5.32\times 10^{-03}$	$8.47\times 10^{-02}$	$8.74\times 10^{-02}$	$8.34\times 10^{-02}$	$8.61\times 10^{-02}$	$3.05\times 10^{-02}$
5.0	$1.95\times 10^{-02}$	$1.99\times 10^{-02}$	$1.91\times 10^{-02}$	$2.00\times 10^{-02}$	$1.72\times 10^{-02}$	$4.47\times 10^{-03}$	$4.60\times 10^{-03}$	$4.27\times 10^{-03}$	$4.66\times 10^{-03}$	$2.90\times 10^{-02}$
10.0	$3.91\times 10^{-03}$	$4.20\times 10^{-03}$	$3.77\times 10^{-03}$	$4.05\times 10^{-03}$	$6.86\times 10^{-02}$	$1.47\times 10^{-03}$	$1.44\times 10^{-03}$	$1.35\times 10^{-03}$	$1.59\times 10^{-03}$	$1.72\times 10^{-02}$
20.0	$2.23\times 10^{-04}$	$3.73\times 10^{-04}$	$1.89\times 10^{-04}$	$2.57\times 10^{-04}$	$4.03\times 10^{-01}$	$3.41\times 10^{-04}$	$3.94\times 10^{-04}$	$2.91\times 10^{-04}$	$3.90\times 10^{-04}$	$1.36\times 10^{-01}$
30.0	$3.46\times 10^{-05}$	$4.43\times 10^{-05}$	$9.03\times 10^{-06}$	$6.02\times 10^{-05}$	$2.18\times 10^{-01}$	$1.52\times 10^{-04}$	$1.49\times 10^{-04}$	$8.20\times 10^{-05}$	$2.22\times 10^{-04}$	$1.80\times 10^{-02}$
40.0	$7.67\times 10^{-07}$	$9.34\times 10^{-03}$	$-4.86\times 10^{-07}$	$2.02\times 10^{-06}$	$1.00\times 10^{+00}$	$4.75\times 10^{-05}$	$2.74\times 10^{-01}$	$3.37\times 10^{-05}$	$6.14\times 10^{-05}$	$1.00\times 10^{+00}$

**Table 5:** Comparing luminance $L(\theta =0.01^{\circ })$ with SWEET and MS for a point light source and for σ={0.01,0.6,0.99} m^−1^, g = {0,0.9} and a set of distances, the mean values of SWEET and MS and the 95% confidence intervals are shown and the relative differences, luminance values are in (W/m^2^/sr), red-colored values are for relatively high discrepancies.

Distance (m)	g = 0.0					g = 0.9				
	Mean SWEET	Mean MS	Inf SWEET	Sup SWEET	Rel Error	Mean SWEET	Mean MS	Inf SWEET	Sup SWEET	Rel Error
σ = 0.01 m^−1^										
0.001	$1.01\times 10^{+06}$	$1.43\times 10^{+04}$	$1.01\times 10^{+06}$	$1.02\times 10^{+06}$	$6.99\times 10^{+01}$	$1.09\times 10^{+06}$	$9.37\times 10^{+04}$	$1.09\times 10^{+06}$	$1.10\times 10^{+06}$	$1.06\times 10^{+01}$
0.5	$1.97\times 10^{+01}$	$1.68\times 10^{+01}$	$1.95\times 10^{+01}$	$1.99\times 10^{+01}$	$1.70\times 10^{-01}$	$1.13\times 10^{+02}$	$1.09\times 10^{+02}$	$1.13\times 10^{+02}$	$1.14\times 10^{+02}$	$3.67\times 10^{-02}$
1.0	$5.63\times 10^{+00}$	$5.28\times 10^{+00}$	$5.58\times 10^{+00}$	$5.68\times 10^{+00}$	$6.50\times 10^{-02}$	$3.41\times 10^{+01}$	$3.35\times 10^{+01}$	$3.40\times 10^{+01}$	$3.42\times 10^{+01}$	$1.84\times 10^{-02}$
5.0	$1.92\times 10^{-02}$	$1.84\times 10^{-02}$	$1.86\times 10^{-02}$	$1.99\times 10^{-02}$	$4.54\times 10^{-02}$	$1.23\times 10^{-01}$	$1.25\times 10^{-01}$	$1.21\times 10^{-01}$	$1.24\times 10^{-01}$	$1.49\times 10^{-02}$
10.0	$5.65\times 10^{-05}$	$6.80\times 10^{-05}$	$4.07\times 10^{-05}$	$7.22\times 10^{-05}$	$1.70\times 10^{-01}$	$4.18\times 10^{-04}$	$4.29\times 10^{-04}$	$3.78\times 10^{-04}$	$4.59\times 10^{-04}$	$2.46\times 10^{-02}$
20.0	$1.50\times 10^{-10}$	$1.49\times 10^{-14}$	$-1.30\times 10^{-10}$	$4.31\times 10^{-10}$	$1.01\times 10^{+04}$	$2.23\times 10^{-10}$	$1.28\times 10^{-12}$	$5.37\times 10^{-11}$	$3.92\times 10^{-10}$	$1.74\times 10^{+02}$
σ=0.6 m^−1^										
0.001	$1.96\times 10^{+06}$	$8.57\times 10^{+05}$	$1.78\times 10^{+06}$	$2.14\times 10^{+06}$	$1.28\times 10^{+00}$	$6.43\times 10^{+06}$	$5.62\times 10^{+06}$	$6.07\times 10^{+06}$	$6.79\times 10^{+06}$	$1.44\times 10^{-01}$
0.5	$1.05\times 10^{+03}$	$1.01\times 10^{+03}$	$1.04\times 10^{+03}$	$1.06\times 10^{+03}$	$3.52\times 10^{-02}$	$6.70\times 10^{+03}$	$6.57\times 10^{+03}$	$6.67\times 10^{+03}$	$6.72\times 10^{+03}$	$1.96\times 10^{-02}$
1.0	$3.14\times 10^{+02}$	$3.18\times 10^{+02}$	$3.11\times 10^{+02}$	$3.17\times 10^{+02}$	$1.08\times 10^{-02}$	$2.02\times 10^{+03}$	$2.01\times 10^{+03}$	$2.02\times 10^{+03}$	$2.03\times 10^{+03}$	$5.43\times 10^{-03}$
5.0	$1.16\times 10^{+00}$	$1.11\times 10^{+00}$	$1.12\times 10^{+00}$	$1.20\times 10^{+00}$	$4.51\times 10^{-02}$	$7.63\times 10^{+00}$	$7.60\times 10^{+00}$	$7.53\times 10^{+00}$	$7.72\times 10^{+00}$	$3.84\times 10^{-03}$
10.0	$4.15\times 10^{-03}$	$4.12\times 10^{-03}$	$3.01\times 10^{-03}$	$5.29\times 10^{-03}$	$7.03\times 10^{-03}$	$2.53\times 10^{-02}$	$2.79\times 10^{-02}$	$2.27\times 10^{-02}$	$2.78\times 10^{-02}$	$9.63\times 10^{-02}$
20.0	$6.05\times 10^{-10}$	$1.11\times 10^{-10}$	$3.17\times 10^{-10}$	$8.92\times 10^{-10}$	$4.45\times 10^{+00}$	$4.99\times 10^{-07}$	$5.04\times 10^{-07}$	$3.77\times 10^{-07}$	$6.22\times 10^{-07}$	$9.58\times 10^{-03}$
σ=0.99 m^−1^										
0.001	$2.40\times 10^{+06}$	$1.41\times 10^{+06}$	$2.12\times 10^{+06}$	$2.68\times 10^{+06}$	$6.98\times 10^{-01}$	$9.80\times 10^{+06}$	$9.27\times 10^{+06}$	$9.21\times 10^{+06}$	$1.04\times 10^{+07}$	$1.20\times 10^{-02}$
0.5	$2.12\times 10^{+02}$	$2.08\times 10^{+02}$	$2.12\times 10^{+02}$	$2.17\times 10^{+02}$	$2.39\times 10^{-02}$	$1.05\times 10^{+03}$	$1.06\times 10^{+03}$	$1.05\times 10^{+03}$	$1.06\times 10^{+03}$	$5.13\times 10^{-03}$
1.0	$6.52\times 10^{+01}$	$6.50\times 10^{+01}$	$6.50\times 10^{+01}$	$6.55\times 10^{+01}$	$7.94\times 10^{-03}$	$3.48\times 10^{+02}$	$3.49\times 10^{+02}$	$3.47\times 10^{+02}$	$3.49\times 10^{+02}$	$2.56\times 10^{-03}$
5.0	$3.75\times 10^{+00}$	$3.69\times 10^{+00}$	$3.71\times 10^{+00}$	$3.79\times 10^{+00}$	$3.56\times 10^{-02}$	$2.19\times 10^{+01}$	$2.18\times 10^{+01}$	$2.16\times 10^{+01}$	$2.21\times 10^{+01}$	$3.10\times 10^{-02}$
10.0	$2.60\times 10^{-02}$	$2.67\times 10^{-02}$	$2.21\times 10^{-02}$	$2.91\times 10^{-02}$	$6.23\times 10^{-03}$	$1.43\times 10^{-01}$	$1.56\times 10^{-01}$	$1.34\times 10^{-01}$	$1.56\times 10^{-01}$	$1.62\times 10^{-02}$
20.0	$5.62\times 10^{-08}$	$6.02\times 10^{-08}$	$5.81\times 10^{-08}$	$6.12\times 10^{-08}$	$6.85\times 10^{-03}$	$5.94\times 10^{-05}$	$5.94\times 10^{-05}$	$5.91\times 10^{-05}$	$5.98\times 10^{-05}$	$1.74\times 10^{-02}$

**Table 6:** Comparing luminance $L(\theta =0^{\circ })$ with SWEET and MS for a rectangular light source and for σ={0.01,0.6,0.99} m^−1^, g = {0,0.9} and a set of distances, the mean values of SWEET and MS and the 95% confidence intervals are shown and the relative differences, luminance values are in (W/m^2^/sr), red-colored values are for relatively high discrepancies, - are for points where MS gives null values.

Distance (m)	g = 0.0					g = 0.9				
	Mean SWEET	Mean MC0	Inf SWEET	Sup SWEET	Rel Error	Mean SWEET	Mean MS	Inf SWEET	Sup SWEET	Rel Error
σ=0.01 m^−1^										
0.001	$9.99\times 10^{-1}$	$9.99\times 10^{-1}$	$9.99\times 10^{-1}$	$9.99\times 10^{-1}$	$9.50\times 10^{-5}$	$9.99\times 10^{-1}$	$9.99\times 10^{-1}$	$9.99\times 10^{-1}$	$9.99\times 10^{-1}$	$9.21\times 10^{-5}$
0.5	$6.08\times 10^{-1}$	$6.06\times 10^{-1}$	$6.08\times 10^{-1}$	$6.08\times 10^{-1}$	$1.98\times 10^{-3}$	$6.09\times 10^{-1}$	$6.06\times 10^{-1}$	$6.09\times 10^{-1}$	$6.09\times 10^{-1}$	$4.78\times 10^{-3}$
1.0	$3.69\times 10^{-1}$	$3.68\times 10^{-1}$	$3.69\times 10^{-1}$	$3.69\times 10^{-1}$	$2.60\times 10^{-3}$	$3.71\times 10^{-1}$	$3.68\times 10^{-1}$	$3.71\times 10^{-1}$	$3.71\times 10^{-1}$	$8.92\times 10^{-3}$
5.0	$6.76\times 10^{-3}$	$6.76\times 10^{-3}$	$6.75\times 10^{-3}$	$6.78\times 10^{-3}$	$4.55\times 10^{-4}$	$7.00\times 10^{-3}$	$6.98\times 10^{-3}$	$6.99\times 10^{-3}$	$7.02\times 10^{-3}$	$2.36\times 10^{-3}$
10.0	$4.63\times 10^{-5}$	$4.42\times 10^{-5}$	$4.49\times 10^{-5}$	$4.76\times 10^{-5}$	$4.62\times 10^{-2}$	$4.79\times 10^{-5}$	$4.68\times 10^{-5}$	$4.66\times 10^{-5}$	$4.93\times 10^{-5}$	$2.40\times 10^{-2}$
20.0	$1.38\times 10^{-11}$	$0.00\times 10^{+0}$	$-3.61\times 10^{-12}$	$3.11\times 10^{-11}$	-	$1.00\times 10^{-10}$	$9.36\times 10^{-12}$	$9.06\times 10^{-11}$	$1.10\times 10^{-10}$	$9.69\times 10^{+0}$
σ=0.6 m^−1^										
0.001	$9.99\times 10^{-1}$	$9.99\times 10^{-1}$	$9.99\times 10^{-1}$	$9.99\times 10^{-1}$	$1.02\times 10^{-4}$	$1.00\times 10^{+0}$	$1.00\times 10^{+0}$	$1.00\times 10^{+0}$	$1.00\times 10^{+0}$	$1.59\times 10^{-4}$
0.5	$6.89\times 10^{-1}$	$6.78\times 10^{-1}$	$6.88\times 10^{-1}$	$6.89\times 10^{-1}$	$1.59\times 10^{-2}$	$8.08\times 10^{-1}$	$8.19\times 10^{-1}$	$8.08\times 10^{-1}$	$8.08\times 10^{-1}$	$1.28\times 10^{-2}$
1.0	$4.51\times 10^{-1}$	$4.39\times 10^{-1}$	$4.51\times 10^{-1}$	$4.51\times 10^{-1}$	$2.77\times 10^{-2}$	$6.46\times 10^{-1}$	$6.37\times 10^{-1}$	$6.46\times 10^{-1}$	$6.46\times 10^{-1}$	$1.45\times 10^{-2}$
5.0	$9.85\times 10^{-3}$	$9.84\times 10^{-3}$	$9.84\times 10^{-3}$	$9.87\times 10^{-3}$	$9.35\times 10^{-4}$	$6.81\times 10^{-2}$	$6.73\times 10^{-2}$	$6.81\times 10^{-2}$	$6.81\times 10^{-2}$	$1.25\times 10^{-2}$
10.0	$6.85\times 10^{-5}$	$6.79\times 10^{-5}$	$6.72\times 10^{-5}$	$6.99\times 10^{-5}$	$9.29\times 10^{-3}$	$1.95\times 10^{-3}$	$1.95\times 10^{-3}$	$1.95\times 10^{-3}$	$1.96\times 10^{-3}$	$9.58\times 10^{-4}$
20.0	$7.33\times 10^{-10}$	$0.00\times 10^{+0}$	$5.89\times 10^{-11}$	$1.41\times 10^{-9}$	-	$8.69\times 10^{-7}$	$1.05\times 10^{-6}$	$7.31\times 10^{-7}$	$1.01\times 10^{-6}$	$1.69\times 10^{-1}$
σ=0.99 m^−1^										
0.001	$1.00\times 10^{+0}$	$1.00\times 10^{+0}$	$1.00\times 10^{+0}$	$1.00\times 10^{+0}$	$2.28\times 10^{-5}$	$1.00\times 10^{+0}$	$1.00\times 10^{+0}$	$1.00\times 10^{+0}$	$1.00\times 10^{+0}$	$9.96\times 10^{-6}$
0.5	$7.88\times 10^{-1}$	$7.79\times 10^{-1}$	$7.88\times 10^{-1}$	$7.88\times 10^{-1}$	$1.11\times 10^{-2}$	$9.78\times 10^{-1}$	$9.74\times 10^{-1}$	$9.78\times 10^{-1}$	$9.78\times 10^{-1}$	$4.00\times 10^{-3}$
1.0	$5.69\times 10^{-1}$	$5.63\times 10^{-1}$	$5.68\times 10^{-1}$	$5.69\times 10^{-1}$	$1.05\times 10^{-2}$	$9.27\times 10^{-1}$	$9.30\times 10^{-1}$	$9.27\times 10^{-1}$	$9.27\times 10^{-1}$	$3.27\times 10^{-3}$
5.0	$1.60\times 10^{-2}$	$1.61\times 10^{-2}$	$1.60\times 10^{-2}$	$1.61\times 10^{-2}$	$8.53\times 10^{-3}$	$1.04\times 10^{-1}$	$1.06\times 10^{-1}$	$1.04\times 10^{-1}$	$1.04\times 10^{-1}$	$1.71\times 10^{-2}$
10.0	$1.12\times 10^{-4}$	$1.17\times 10^{-4}$	$1.12\times 10^{-4}$	$1.13\times 10^{-4}$	$4.40\times 10^{-2}$	$3.22\times 10^{-3}$	$3.28\times 10^{-3}$	$3.21\times 10^{-3}$	$3.23\times 10^{-3}$	$1.99\times 10^{-2}$
20.0	$1.23\times 10^{-7}$	$3.85\times 10^{-7}$	$1.78\times 10^{-8}$	$2.28\times 10^{-7}$	-	$4.26\times 10^{-6}$	$4.84\times 10^{-6}$	$2.62\times 10^{-6}$	$5.91\times 10^{-6}$	$1.37\times 10^{-1}$

**Table 7:** Comparison of luminance $L(\theta =0.01^{\circ })$ from SWEET and MS for a configuration with two point light sources and for σ={0.01,0.6,0.99} m^−1^, g = {0,0.9} and a set of distances, the mean values of SWEET and MS and the 95% confidence intervals are shown and the relative differences, luminance values are in (W/m^2^/sr), red-colored values are for relatively high discrepancies.

Distance (m)	g = 0.0					g = 0.9				
	Mean SWEET	Mean MC0	Inf SWEET	Sup SWEET	Rel Error	Mean SWEET	Mean MC0	Inf SWEET	Sup SWEET	Rel Error
$\sigma =0.01\;m^{-1}$										
0.001	$3.12\times 10^{-4}$	$3.09\times 10^{-4}$	$3.12\times 10^{-4}$	$3.12\times 10^{-4}$	$7.98\times 10^{-3}$	$1.67\times 10^{-5}$	$1.66\times 10^{-5}$	$1.66\times 10^{-5}$	$1.67\times 10^{-5}$	$3.39\times 10^{-3}$
0.5	$3.94\times 10^{-4}$	$3.91\times 10^{-4}$	$3.94\times 10^{-4}$	$3.94\times 10^{-4}$	$9.33\times 10^{-3}$	$3.57\times 10^{-5}$	$3.56\times 10^{-5}$	$3.57\times 10^{-5}$	$3.58\times 10^{-5}$	$1.92\times 10^{-3}$
1.0	$3.54\times 10^{-4}$	$3.51\times 10^{-4}$	$3.54\times 10^{-4}$	$3.54\times 10^{-4}$	$6.65\times 10^{-3}$	$5.71\times 10^{-5}$	$5.70\times 10^{-5}$	$5.71\times 10^{-5}$	$5.71\times 10^{-5}$	$2.45\times 10^{-3}$
5.0	$1.15\times 10^{-5}$	$1.15\times 10^{-5}$	$1.15\times 10^{-5}$	$1.15\times 10^{-5}$	$1.83\times 10^{-4}$	$2.20\times 10^{-5}$	$2.20\times 10^{-5}$	$2.20\times 10^{-5}$	$2.20\times 10^{-5}$	$1.28\times 10^{-3}$
10.0	$8.45\times 10^{-8}$	$8.46\times 10^{-8}$	$8.36\times 10^{-8}$	$8.54\times 10^{-8}$	$1.11\times 10^{-3}$	$4.29\times 10^{-7}$	$4.35\times 10^{-7}$	$4.28\times 10^{-7}$	$4.30\times 10^{-7}$	$1.44\times 10^{-2}$
20.0	$3.37\times 10^{-12}$	$3.31\times 10^{-14}$	$-1.71\times 10^{-13}$	$6.92\times 10^{-12}$	$1.01\times 10^{2}$	$3.75\times 10^{-11}$	$4.10\times 10^{-12}$	$3.45\times 10^{-11}$	$4.05\times 10^{-11}$	$8.16\times 10^{0}$
$\sigma =0.6\;m^{-1}$										
0.001	$4.78\times 10^{-2}$	$4.75\times 10^{-2}$	$4.77\times 10^{-2}$	$4.80\times 10^{-2}$	$7.69\times 10^{-3}$	$2.59\times 10^{-3}$	$2.45\times 10^{-3}$	$2.55\times 10^{-3}$	$2.64\times 10^{-3}$	$5.75\times 10^{-2}$
0.5	$5.88\times 10^{-2}$	$5.80\times 10^{-2}$	$5.86\times 10^{-2}$	$5.90\times 10^{-2}$	$1.32\times 10^{-2}$	$5.66\times 10^{-3}$	$5.43\times 10^{-3}$	$5.41\times 10^{-3}$	$5.91\times 10^{-3}$	$4.17\times 10^{-2}$
1.0	$5.43\times 10^{-2}$	$5.49\times 10^{-2}$	$5.42\times 10^{-2}$	$5.44\times 10^{-2}$	$1.06\times 10^{-2}$	$9.68\times 10^{-3}$	$9.65\times 10^{-3}$	$9.53\times 10^{-3}$	$9.83\times 10^{-3}$	$3.65\times 10^{-3}$
5.0	$2.48\times 10^{-3}$	$2.46\times 10^{-3}$	$2.42\times 10^{-3}$	$2.54\times 10^{-3}$	$5.97\times 10^{-3}$	$1.36\times 10^{-2}$	$1.33\times 10^{-2}$	$1.35\times 10^{-2}$	$1.37\times 10^{-2}$	$1.96\times 10^{-2}$
10.0	$2.27\times 10^{-5}$	$2.26\times 10^{-5}$	$2.22\times 10^{-5}$	$2.32\times 10^{-5}$	$2.76\times 10^{-3}$	$8.27\times 10^{-4}$	$8.73\times 10^{-4}$	$8.05\times 10^{-4}$	$8.48\times 10^{-4}$	$5.32\times 10^{-2}$
20.0	$1.36\times 10^{-9}$	$2.12\times 10^{-10}$	$5.04\times 10^{-10}$	$2.21\times 10^{-9}$	$5.40\times 10^{0}$	$9.75\times 10^{-7}$	$5.58\times 10^{-7}$	$7.90\times 10^{-7}$	$1.16\times 10^{-6}$	$7.47\times 10^{-1}$
$\sigma =0.99\;m^{-1}$										
0.001	$3.01\times 10^{-1}$	$2.98\times 10^{-1}$	$3.00\times 10^{-1}$	$3.01\times 10^{-1}$	$7.75\times 10^{-3}$	$1.21\times 10^{-2}$	$1.08\times 10^{-2}$	$1.17\times 10^{-2}$	$1.26\times 10^{-2}$	$1.28\times 10^{-1}$
0.5	$3.46\times 10^{-1}$	$3.37\times 10^{-1}$	$3.44\times 10^{-1}$	$3.49\times 10^{-1}$	$2.62\times 10^{-2}$	$2.18\times 10^{-2}$	$2.01\times 10^{-2}$	$2.08\times 10^{-2}$	$2.28\times 10^{-2}$	$8.25\times 10^{-2}$
1.0	$3.34\times 10^{-1}$	$3.30\times 10^{-1}$	$3.32\times 10^{-1}$	$3.37\times 10^{-1}$	$1.35\times 10^{-2}$	$3.65\times 10^{-2}$	$3.42\times 10^{-2}$	$3.52\times 10^{-2}$	$3.78\times 10^{-2}$	$6.43\times 10^{-2}$
5.0	$6.66\times 10^{-2}$	$6.55\times 10^{-2}$	$6.59\times 10^{-2}$	$6.71\times 10^{-2}$	$2.05\times 10^{-2}$	$1.74\times 10^{-1}$	$1.70\times 10^{-1}$	$1.72\times 10^{-1}$	$1.75\times 10^{-1}$	$2.43\times 10^{-2}$
10.0	$1.34\times 10^{-3}$	$1.34\times 10^{-3}$	$1.32\times 10^{-3}$	$1.35\times 10^{-3}$	$4.97\times 10^{-3}$	$2.43\times 10^{-2}$	$2.54\times 10^{-2}$	$2.37\times 10^{-2}$	$2.50\times 10^{-2}$	$4.51\times 10^{-2}$
20.0	$2.96\times 10^{-8}$	$4.89\times 10^{-9}$	$1.26\times 10^{-8}$	$5.66\times 10^{-8}$	$8.42\times 10^{0}$	$3.89\times 10^{-4}$	$2.27\times 10^{-4}$	$3.00\times 10^{-4}$	$4.41\times 10^{-4}$	$4.15\times 10^{-1}$

## Data Availability

The raw data supporting the conclusions of this article will be made available by the authors on request.

## Abbreviations

The following abbreviations are used in this manuscript:

SWEET	Simulating WEather for intElligent Transportation systems
PAVIN	Plateforme d’Auvergne Véhicules Intelligents
ADAS	Advanced Driver Assistance Systems
ADS	Automated Driving System
LiDAR	Light Detection And Ranging
MC	Monte Carlo
RADAR	RAdio Detection And Ranging
RTE	Radiative Transfer Equation
MIS	Multiple Importance Sampling
DSD	Droplet Size Distribution
MOR	Meteorological Optical Range
MS	Mitsuba
MC0	Steven’s MC code ([44], available online)
BRDF	Bidirectional Reflectance Distribution Function
BTDF	Bidirectional Transmission Distribution Function
BSDF	Bidirectional Scattering Distribution Function
